# Therapeutic targeting of YY1/MZF1 axis by MZF1-uPEP inhibits aerobic glycolysis and neuroblastoma progression

**DOI:** 10.7150/thno.37383

**Published:** 2020-01-01

**Authors:** Erhu Fang, Xiaojing Wang, Jianqun Wang, Anpei Hu, Huajie Song, Feng Yang, Dan Li, Wenjing Xiao, Yajun Chen, Yanhua Guo, Yang Liu, Hongjun Li, Kai Huang, Liduan Zheng, Qiangsong Tong

**Affiliations:** 1 Department of Pediatric Surgery, Union Hospital, Tongji Medical College, Huazhong University of Science and Technology, 1277 Jiefang Avenue, Wuhan 430022, Hubei Province, P. R. China.; 2 Clinical Center of Human Genomic Research, Union Hospital, Tongji Medical College, Huazhong University of Science and Technology, 1277 Jiefang Avenue, Wuhan 430022, Hubei Province, P. R. China.; 3 Department of Pathology, Union Hospital, Tongji Medical College, Huazhong University of Science and Technology, 1277 Jiefang Avenue, Wuhan 430022, Hubei Province, P. R. China.

**Keywords:** aerobic glycolysis, myeloid zinc finger 1, tumor progression, upstream open reading frame, Yin Yang 1.

## Abstract

As a hallmark of metabolic reprogramming, aerobic glycolysis contributes to tumorigenesis and aggressiveness. However, the mechanisms and therapeutic strategies regulating aerobic glycolysis in neuroblastoma (NB), one of leading causes of cancer-related death in childhood, still remain elusive.

**Methods**: Transcriptional regulators and their downstream glycolytic genes were identified by a comprehensive screening of publicly available datasets. Dual-luciferase, chromatin immunoprecipitation, real-time quantitative RT-PCR, western blot, gene over-expression or silencing, co-immunoprecipitation, mass spectrometry, peptide pull-down assay, sucrose gradient sedimentation, seahorse extracellular flux, MTT colorimetric, soft agar, matrigel invasion, and nude mice assays were undertaken to explore the biological effects and underlying mechanisms of transcriptional regulators in NB cells. Survival analysis was performed by using log-rank test and Cox regression assay.

**Results**: Transcription factor myeloid zinc finger 1 (MZF1) was identified as an independent prognostic factor (hazard ratio=2.330, 95% confidence interval=1.021 to 3.317), and facilitated glycolysis process through increasing expression of hexokinase 2 (*HK2*) and phosphoglycerate kinase 1 (*PGK1*). Meanwhile, a 21-amino acid peptide encoded by upstream open reading frame of *MZF1*, termed as MZF1-uPEP, bound to zinc finger domain of Yin Yang 1 (YY1), resulting in repressed transactivation of YY1 and decreased transcription of *MZF1* and downstream genes *HK2* and *PGK1*. Administration of a cell-penetrating MZF1-uPEP or lentivirus over-expressing MZF1-uPEP inhibited the aerobic glycolysis, tumorigenesis and aggressiveness of NB cells. In clinical NB cases, low expression of MZF1-uPEP or high expression of *MZF1*, *YY1*, *HK2*, or *PGK1* was associated with poor survival of patients.

**Conclusions**: These results indicate that therapeutic targeting of *YY1*/*MZF1* axis by MZF1-uPEP inhibits aerobic glycolysis and NB progression.

## Introduction

Neuroblastoma (NB) is the most common extracranial solid malignancy in pediatric population, and accounts for approximately 15% of all childhood cancer deaths 1. Despite advances in molecular mechanisms and multimodal therapy 2, 3, the clinical course of high-risk NB cases still remains unfavorable, and is featured by rapid progression and high mortality 1. As a hallmark of metabolic reprogramming, even in the presence of oxygen, tumor cells uptake and convert a large amount of glucose into lactic acid to support their tumorigenecity and aggressiveness, which is known as aerobic glycolysis or Warburg effect 4-7. High rates of glycolysis are consistently observed in most of tumors, accompanied by up-regulation of glycolytic enzymes such as hexokinase 2 (*HK2*), phosphoglycerate kinase 1 (*PGK1*), and enolase 1 (*ENO1*) 8, 9. Meanwhile, small organic molecules, such as 3-bromopyruvate or 2-deoxyglucose (2-DG), are able to inhibit aerobic glycolysis and exhibit therapeutic potential for repressing tumor progression 10, 11. Thus, it is important to investigate the mechanisms and therapeutic strategies for aerobic glycolysis during tumor progression.

Recent studies show that aerobic glycolysis is driven by activation of oncogenes or inactivation of tumor suppressors. For example, hypoxia inducible factor 1 alpha, a key mediator of hypoxic response, contributes to aerobic glycolysis by up-regulating glucose transporters 1 (*GLUT1*) and lactate dehydrogenase A (*LDHA*) 12. Onocgenic c-Myc facilitates glycolysis process through inducing *HK2* and* LDHA* expression 13, 14. Meanwhile, p53 represses aerobic glycolysis through reducing promoter activity of *GLUT1* and *GLUT4* 15. Long noncoding RNA *LINC01554* inhibits aerobic glycolysis via promoting degradation of pyruvate kinase isozyme M2 (PKM2) in hepatocellular carcinoma cells 7. However, the mechanisms regulating the expression of glycolytic genes in NB still remain to be determined.

In this study, through an integrative screening approach, we identify myeloid zinc finger 1 (*MZF1*) and its upstream open reading frame (uORF)-derived peptide (uPEP) as crucial regulators of aerobic glycolysis and NB progression. We demonstrate that *MZF1* is up-regulated in NB tissues and cells, and facilitates the aerobic glycolysis, growth, and aggressiveness of NB cells by up-regulating *HK2* and *PGK1*. Meanwhile, MZF1-uPEP interacts with Yin Yang 1 (YY1) to repress its transactivation, resulting in transcriptional inhibition of *MZF1* and downstream glycolytic genes. Pre-clinically, administration of a cell-penetrating MZF1-uPEP or lentivirus over-expressing MZF1-uPEP significantly suppresses aerobic glycolysis, tumorigenesis and aggressiveness, indicating the crucial roles of MZF1-uPEP in repressing *YY1*/*MZF1* axis during NB progression.

## Methods

### Cell culture

Human non-transformed mammary epithelial MCF 10A (CRL-10317) cells, embryonic kidney HEK293 (CRL-1573) cells, NB cell lines SH-SY5Y (CRL-2266), SK-N-AS (CRL-2137), BE(2)-C (CRL-2268), and IMR-32 (CCL-127), and cervical cancer HeLa (CCL-2) cells were purchased from American Type Culture Collection (Rockville, MD). Cell lines were authenticated by short tandem repeat profiling, and used within 6 months after resuscitation of frozen aliquots. Mycoplasma contamination was regularly examined using Lookout Mycoplasma PCR Detection Kit (Sigma, St. Louis, MO). Cells were maintained in Dulbecco's modified Eagle's medium (DMEM) supplemented with 10% fetal bovine serum (Gibco, Grand Island, NY) at 37°C in a humidified atmosphere of 5% CO_2_, and treated with 2-DG, insulin-like growth factor 1 (IGF1), or LY294002 as indicated (Sigma).

### Real-time quantitative RT-PCR (qRT-PCR)

Total RNA was isolated with RNeasy Mini Kit (Qiagen Inc., Valencia, CA). Reverse transcription reactions were conducted with Transcriptor First Strand cDNA Synthesis Kit (Roche, Indianapolis, IN). Real-time PCR was performed with SYBR Green PCR Master Mix (Applied Biosystems, Foster City, CA) and primers (Table S1).

### Western blot

The peptide corresponding to MZF-uPEP (METRWGTDGVLMTAVIGAGSC) was synthesized, and coupled to keyhole limpet hemocyanin using chemical crosslinker glutaraldehyde. Rabbit anti-MZF1-uPEP polyclonal antibody was prepared by immunizing New Zealand rabbit with synthesized peptide, purified by persulfate, Sephadex G25 and DEAE-Sephadex G100 (ABclonal Biotechnology Co., Ltd, Wuhan, China), and validated by antigen peptide or fusion protein recognition. Tissue or cellular protein was extracted with 1× cell lysis buffer (Promega, Madison, WI). Western blot was performed as previously described 16-20, with antibodies for MZF1 (ab64866), HK2 (ab104836), PGK1 (ab113687), phosphorylated AKT (p-AKT, ab38449), AKT (ab8805, Abcam Inc., Cambridge, MA), YY1 (D3D4Q, Cell Signaling Technology, Inc., Danvers, MA), upstream transcription factor 2 (USF2, ab125184), GFP (ab290), Flag (ab1162), Myc (ab9106), or β-actin (ab6276, Abcam Inc.).

### Luciferase reporter assay

The 5'-untranslated region (5'-UTR, 561 bp) of *MZF1* and promoters of *MZF1* (-1530/+30)*, HK2* (-1813/+424), or *PGK1* (-882/+246) were amplified from genomic DNA by PCR (Table S2) and subcloned into pGL3-Basic (Promega). Luciferase reporter for analyzing transactivation of *YY1* was established by annealing complementary oligonucleotides containing four canonical binding sites (Table S2) and inserting into pGL3-Basic (Promega). Mutation of YY1 or MZF1 binding site was performed with GeneTailorTM Site-Directed Mutagenesis System (Invitrogen, Carlsbad, CA) and PCR primers (Table S2). Dual-luciferase assay was performed according to manufacturer's instructions (Promega) 16, 17, 19, 20.

### Chromatin immunoprecipitation (ChIP)

ChIP assay was performed according to instructions of EZ-ChIP kit (Upstate Biotechnology, Temacula, CA) 16-18. Real-time quantitative PCR (qPCR) was performed with SYBR Green PCR Master Mix (Applied Biosystems) and primers (Table S1).

### Gene over-expression and knockdown

Human *MZF1* coding sequence (CDS, 2205 bp), *MZF1* cDNA (2920 bp), *YY1* cDNA (1245 bp) and corresponding truncations were obtained from NB tissues by PCR primers (Table S2), and inserted into pcDNA3.1 (Invitrogen), pEGFP-N1, pCMV-3Tag-1C, pCMV-C-Flag, pCMV-N-MYC (Addgene, Cambridge, MA), or lentiviral expression vector CV186 (Genechem Co., Ltd, Shanghai, China), respectively. Human *HK2* and *PGK1* expression vectors were obtained from Genechem Co., Ltd. Mutation and frame-shift deletion of *GFP* or *MZF1-uORF* was prepared with GeneTailor^TM^ Site-Directed Mutagenesis System (Invitrogen) and primers (Table S2). Oligonucleotides encoding short hairpin RNAs (shRNAs) specific for *MZF1*, *HK2*, *PGK1*, *MZF1-uORF*, or *YY1* (Table S3) were subcloned into GV298 (Genechem Co., Ltd). Single guide RNAs (sgRNAs) were designed using Guide Design Resources (http://crispr.mit.edu) to target upstream or downstream region relative to transcription start site of *MZF1* (Table S2), and inserted into dCas9-VPR or dCas9-BFP-KRAB (Addgene), respectively. Stable cell lines were screened by administration of neomycin or puromycin (Invitrogen).

### Rescue of target gene expression

To restore target gene expression induced by *MZF1* or *MZF1-uORF*, tumor cells were transfected with shRNAs targeting *HK2* and *PGK1*, or *YY1* expression vector. To rescue gene expression altered by knockdown of *MZF1* or *MZF1-uORF*, *HK2* and *PGK1* expression vectors or shRNAs specific for *YY1* (Table S3) were transfected into tumor cells with Lipofectamine 3000 (Life Technologies, Inc., Gaithersburg, MD).

### Lentiviral packaging

Lentiviral vectors were co-transfected with packaging plasmids psPAX2 and pMD2G (Addgene) into HEK293T cells. Infectious lentivirus was harvested and filtered through 0.45 μm PVDF filters. Recombinant lentiviruses were concentrated 100-fold by ultracentrifugation (2 hrs at 120,000 g).

### Fluorescence immunocytochemical staining

Tumor cells were plated on coverslips and incubated with antibodies specific for YY1 (ab109237, Abcam Inc., 1:300 dilution) or MZF1-uPEP (1:200 dilution). Then, cells were incubated with Alexa Fluor 594 goat anti-rabbit IgG or Alexa Fluor 488 goat anti-rabbit IgG (1:1000 dilutions), and stained with 4',6-diamidino-2-phenylindole (DAPI, 300 nmol/L).

### Co-immunoprecipitation (co-IP) and mass spectrometry

Co-IP was performed as previously described 17, 18, 20, with antibodies for Flag (ab1162) or Myc (ab9106, Abcam, Inc.). Bead-bound proteins were released, separated using SDS-PAGE, and analyzed by Coomassie blue staining, western blot, or mass spectrometry (Wuhan Institute of Biotechnology, Wuhan, China).

### Design and synthesis of cell-penetrating peptides

Cell-penetrating peptide of MZF1-uPEP was designed and synthesized (ChinaPeptides Co. Ltd, Shanghai, China). The 11-amino acid long peptide (YGRKKRRQRRR) from Tat protein transduction domain served as a cell-penetrating peptide. Thus, inhibitory peptides were chemically synthesized by linking with biotin-labeled cell-penetrating peptide at N-terminus and conjugating with fluorescein isothiocyanate (FITC) at C-terminus, with purity larger than 95%.

### Biotin-labeled peptide pull-down assay

Cellular proteins were isolated using 1× cell lysis buffer (Promega), and incubated with biotin-labeled peptide at 4°C overnight. Then, incubation of cell lysates with streptavidin-agarose was undertaken at 4°C for 2 hrs. Beads were extensively washed, and proteins pulled down were measured by western blot assay.

### Aerobic glycolysis and seahorse extracellular flux assays

Cellular glucose uptake, lactate production, and adenosine triphosphate (ATP) levels were detected as previously described 21. Extracellular acidification rate (ECAR) and oxygen consumption rate (OCR) were measured under basal conditions and in response to glucose (10 mmol·L^-1^), oligomycin (2 μmol·L^-1^), and 2-DG (100 mmol·L^-1^) using a Seahorse Biosciences XFe24 Flux Analyzer (North Billerica, MA) 22.

### Sucrose gradient sedimentation

Tumor cells were treated with 100 μg/ml of cycloheximide (Sigma) for 5-10 min. Cell extracts were layered on top of 15-30% (w/v) linear sucrose gradient. After centrifugation at 40,000 ×g for 2 hrs at 4°C, fractions were collected using a piston-gradient fractionator (Biocomp, Fredericton, Canada). The polysome profiles were monitored by absorbance of light with a wavelength of 260 nm (A260). The polysome-bound transcripts were extracted and detected by real-time qRT-PCR.

### *In vitro* cell viability, growth, and invasion assays

The 2-(4,5-dimethyltriazol-2-yl)-2,5-diphenyl tetrazolium bromide (MTT, Sigma) colorimetric 18, soft agar 16-20 and matrigel (BD Matrigel^TM^ Matrix, BD Biosciences, Franklin Lakes, NJ) invasion 16-20, 23 assays for measuring the viability, growth, and invasion capability of tumor cells were conducted as previously described.

### *In vivo* tumorigenesis and aggressiveness assays

All animal experiments were carried out in accordance with NIH Guidelines for the Care and Use of Laboratory Animals, and approved by the Animal Care Committee of Tongji Medical College (approval number: Y20080290). *In vivo* tumor growth (1×10^6^ tumor cells per mouse) and experimental metastasis (0.4×10^6^ tumor cells per mouse) studies were performed with blindly randomized four-week-old female BALB/c nude mice as previously described 16-20. For *in vivo* therapeutic studies, tumor cells (1×10^6^ or 0.4×10^6^) were injected into dorsal flanks or tail vein of nude mice, respectively. One week later, mice were blindly randomized and treated by tail vein injection of synthesized cell-penetrating peptide (ChinaPeptides, Shanghai, China) or lentivirus-mediated *MZF1-uORF* (1×10^7^ plaque-forming units in 100 μl phosphate buffer saline). Animals were imaged using the In-Vivo Xtreme II small animal imaging system (Bruker Corporation, Billerica, MA).

### Patient tissue samples

The Institutional Review Board of Tongji Medical College approved the human tissue study (approval number: 2011-S085). All procedures were undertaken in accordance with guidelines set forth by Declaration of Helsinki. Written informed consent was obtained from all legal guardians of patients. Patients with a history of preoperative chemotherapy or radiotherapy were excluded. Human normal dorsal root ganglia tissues were collected from therapeutic abortion. Fresh specimens were collected at surgery, validated by pathological diagnosis, and stored at -80°C.

### Immunohistochemistry

Immunohistochemical staining was performed as previously described 16-18, with antibodies specific for Ki-67 (ab92742; 1:100 dilution), MZF1-uPEP (1:200 dilution), YY1 (ab38422; 1:200 dilution), or MZF1 (ab64866, Abcam Inc.; 1:200 dilution). The immunostaining specificity was validated by neutralizing antigen peptide incubation and IgG isotype control. The reactivity degree was assessed by at least two pathologists without knowledge of tumor groups.

### Statistical analysis

All data were shown as mean ± standard error of the mean (SEM). Cutoff values were determined by average gene expression levels. Student's *t* test, one-way analysis of variance (ANOVA), and χ^2^ analysis were applied to compare the difference in tumor cells or tissues. Fisher's exact test was applied to analyze the statistical significance of overlap between two gene lists. Pearson's correlation coefficient was applied for analyzing the relationship among gene expression. Log-rank test and Cox regression analysis were used to assess survival difference and hazard ratio. All statistical tests were two-sided and considered statistically significant when false discovery rate-corrected *P* values less than 0.05.

## Results

### *MZF1* facilitates the transcription of glycolytic genes in NB

**Figure 1:**
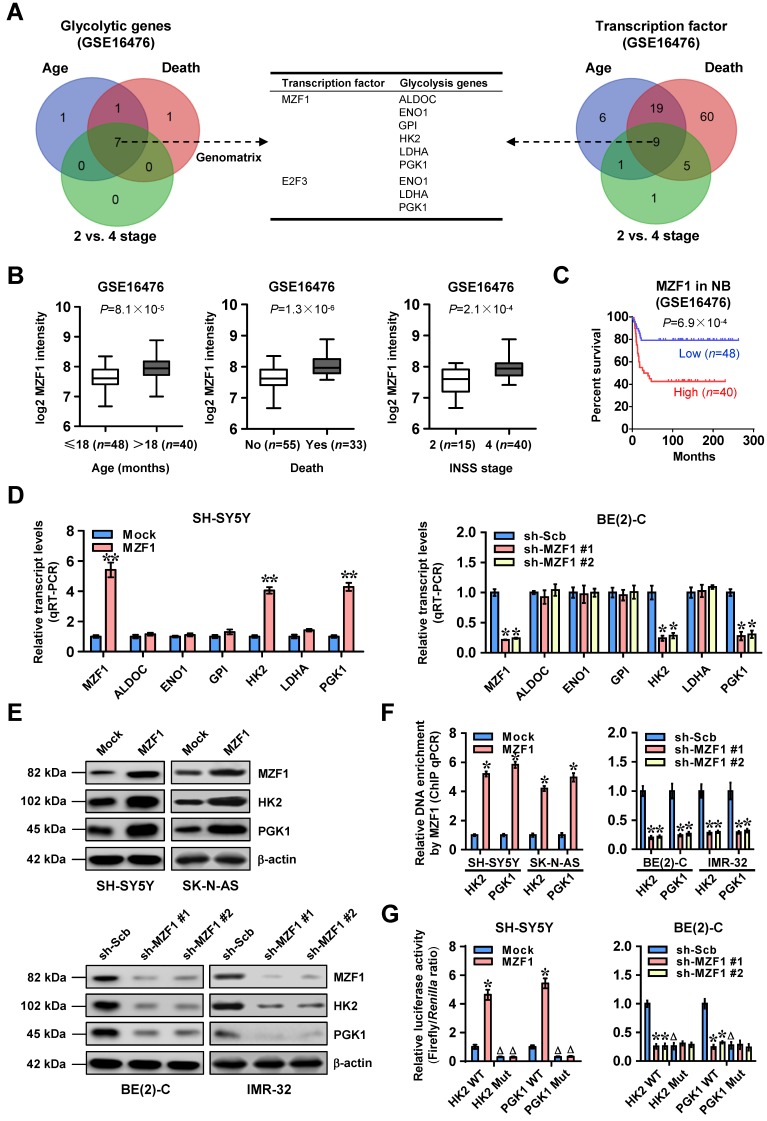
***MZF1* facilitates the transcription of glycolytic genes in NB. (A)** Venn diagram indicating the identification of glycolytic genes (left panel) and transcription factors (right panel) differentially expressed in 88 NB cases (GSE16476) with various status of age, death, and INSS stages, and the over-lapping analysis with potential transcription factors regulating glycolytic genes revealed by Genomatix program. The middle panel showing the potential transcription factors regulating expression of glycolytic genes. **(B)** Mining of a public microarray dataset (GSE16476) revealing the *MZF1* levels in NB tissues with different status of age, death, or INSS stages. **(C)** Kaplan-Meier curve showing overall survival of 88 NB patients (GSE16476) with high or low* MZF1* expression (cutoff value=226.6). **(D** and **E)** Real-time qRT-PCR (D, normalized to β-actin, *n*=4) and western blot (E) assays indicating the transcript and protein levels of *MZF1*, *ALDOC*, *ENO1*, *GPI*, *HK2*, *LDHA*, or *PGK1* in SH-SY5Y and BE(2)-C cells stably transfected with empty vector (mock), *MZF1*, scramble shRNA (sh-Scb), or sh-MZF1.** (F)** ChIP and qPCR assays showing the binding of *MZF1* to promoters of *HK2* and *PGK1* in SH-SY5Y, SK-N-AS, BE(2)-C, and IMR-32 cells stably transfected with mock, *MZF1*, sh-Scb, or sh-MZF1 (*n*=4). **(G)** Dual-luciferase assay indicating the promoter activity of *HK2* and *PGK1* with wild-type (WT) or mutant (Mut) MZF1 binding site in SH-SY5Y and BE(2)-C cells stably transfected with mock, *MZF1*, sh-Scb, or sh-MZF1 (*n*=6). Fisher's exact test for over-lapping analysis in **A**. Student's *t* test compared the difference in** B**. Log-rank test for survival comparison in **C**. Student's *t* test and ANOVA compared the difference in **D**,** F** and **G**. * *P*<0.05, ** *P*<0.01 vs. mock or sh-Scb. ^Δ^ *P*<0.05 vs. WT. Bars are means and whiskers (min to max) in **B**. Data are shown as mean ± s.e.m. (error bars) and representative of three independent experiments in **D**-**G**.

To investigate transcriptional regulators of glycolytic gene expression and tumor progression, we performed comprehensive analysis of a public dataset of 88 NB cases (GSE16476) 24, and identified 9, 9, and 7 glycolytic genes (*P*<0.05) differentially expressed in NB specimens with varied status of age, death, and international neuroblastoma staging system (INSS) stages, respectively (Figure 1A). Based on over-lapping analysis of these results (*P*<0.001), 7 genes were found to be consistently associated with age, death, and advanced INSS stage of NB (Figure 1A and Table S4). Similarly, we also found 9 transcription factors consistently associated with these clinical features of 88 NB cases (Figure 1A and Table S4), which were subjective to further over-lapping analysis with potential transcription factors regulating all of 7 genes revealed by Genomatix program (http://www.genomatix.de). The results revealed that two transcription factors, MZF1 and E2F transcription factor 3 (E2F3), might regulate expression of these glycolytic genes (Figure 1A). Among them, MZF1 was top transcription factor with six potential targets (Figure S1) and chosen for further study. Notably, *MZF1* was highly expressed in NB tissues with elder age (*P*=8.1×10^-5^), death (*P*=1.3×10^-6^), or advanced INSS stage (*P*=2.1×10^-4^), and was associated with poor survival of patients (*P*=6.9×10^-4^ and *P*=5.8×10^-3^, Figure 1B-C, and Figure S2A) as an independent prognostic factor (hazard ratio=2.330, 95% confidence interval=1.021 to 3.317, *P*=0.044). In addition, higher *MZF1* expression was observed in NB cell lines, than that in normal dorsal root ganglia (Figure S2B). To further elucidate the effects of *MZF1* on glycolytic gene expression, we chose SH-SY5Y, SK-N-AS, BE(2)-C, and IMR-32 (with low and high *MZF1* levels, respectively) cells as models. Sable over-expression or knockdown of *MZF1* increased and decreased the levels of *HK2* or *PGK1*, but not of fructose-bisphosphate C (*ALDOC*), *ENO1*, glucose-6-phosphate isomerase (*GPI*), or *LDHA*, in these NB cells, respectively (Figure 1D-E and Figure S2C). The MZF1 enrichment on promoters of *HK2* and *PGK1* was increased and decreased by stable over-expression or knockdown of *MZF1*, respectively (Figure 1F). Ectopic expression or knockdown of *MZF1* facilitated and inhibited the promoter activity of *HK2* and *PGK1* in SH-SY5Y, SK-N-AS, BE(2)-C, and IMR-32 cells, respectively, while mutation of MZF1 binding site abolished these effects (Figure 1G and Figure S2D). Consistently, mining of public datasets (GSE16476 and GSE62564) revealed that *HK2* (*P*=3.0×10^-10^ and* P*=2.4×10^-16^) or *PGK1* (*P*=5.3×10^-8^ and* P*=3.0×10^-23^) levels were associated with poor survival of NB patients (Figure S2E), and were positively correlated with those of *MZF1* (*R*=0.498, *P*=7.9×10^-7^; *R*=0.408, *P*=8.0×10^-5^; Figure S2F). High expression of *MZF1*, *HK2*, or *PGK1* was also associated with poor survival of patients with breast cancer, endometrial carcinoma, glioma, head and neck carcinoma, lung cancer, lymphoma, pancreatic cancer, or renal clear cell carcinoma (Figure S3). These data indicated that transcription factor MZF1 facilitated the expression of glycolytic genes in NB.

### *MZF1* promotes NB progression via facilitating aerobic glycolysis

**Figure 2:**
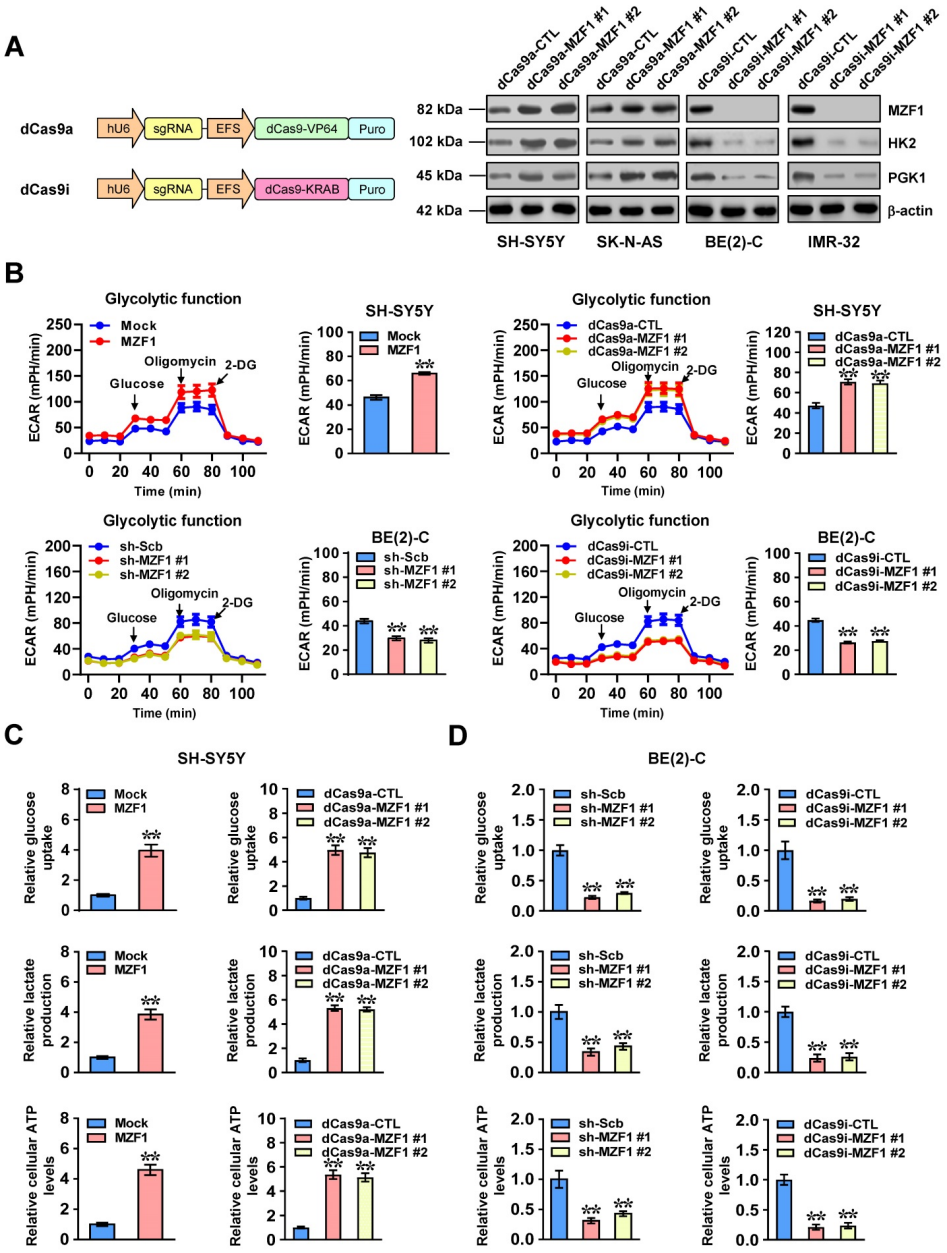
***MZF1* promotes the aerobic glycolysis of NB cells. (A)** Western blot assay indicating the expression of MZF1, HK2, and PGK1 in SH-SY5Y, SK-N-AS, BE(2)-C, and IMR-32 cells stably transfected with dCas9a control (dCas9a-CTL), dCas9a-MZF1, dCas9i control (dCas9i-CTL), or dCas9i-MZF1. **(B)** Seahorse tracing curves (left panel) and ECAR bars (right panel) of SH-SY5Y and BE(2)-C cells stably transfected with empty vector (mock), *MZF1*, scramble shRNA (sh-Scb), sh-MZF1, dCas9a-CTL, dCas9a-MZF1, dCas9i-CTL, or dCas9i-MZF1, and those treated with glucose (10 mmol·L^-1^), oligomycin (2 μmol·L^-1^), or 2-deoxyglucose (2-DG, 100 mmol·L^-1^) at indicated (4 replicates for each point). **(C** and** D)** Glucose uptake, lactate production, and ATP levels in SH-SY5Y (C) and BE(2)-C (D) cells stably transfected with mock, *MZF1*, sh-Scb, sh-MZF1, dCas9a-CTL, dCas9a-MZF1, dCas9i-CTL, or dCas9i-MZF1 (*n*=4). Student's *t* test and ANOVA compared the difference in **B**-**D**. ** *P*<0.01 vs. mock, sh-Scb, dCas9a-CTL, or dCas9i-CTL. Data are shown as mean ± s.e.m. (error bars) and representative of three independent experiments in **A**-**D**.

To characterize the functional roles of *MZF1* in NB cells, we applied dCas9-based clustered regularly interspaced short palindromic repeats (CRISPR) 25 to activate or repress expression of *MZF1*. As shown in Figure 2A, transfection of two independent dCas9a-MZF1 or dCas9i-MZF1 resulted in efficient over-expression or silencing of *MZF1* in NB cells, respectively. Stable transfection- or dCas9a-induced up-regulation of *MZF1* increased the ECAR, an indicator of glycolysis, in SH-SY5Y and SK-N-AS cells, while shRNA- or dCas9i-induced knockdown of *MZF1* significantly attenuated glycolytic process in BE(2)-C and IMR-32 cells (Figure 2B and Figure S4A). Meanwhile, OCR was reduced and enhanced in NB cells with over-expression or knockdown of *MZF1*, respectively (Figure S4B). Accordingly, ectopic expression or knockdown of *MZF1* increased and decreased the glucose uptake, lactate production, and ATP levels in NB cells, suggesting facilitated and reduced glycolysis, respectively (Figure 2C-D and Figure S4C-D).

To explore the roles of *HK2* and *PGK1* in *MZF1*-facilitated aerobic glycolysis, shRNAs or expression vectors of *HK2* and *PGK1* were transfected into SH-SY5Y and BE(2)-C cells to restore their expression, glucose uptake, lactate production, and ATP levels altered by stable over-expression or knockdown of *MZF1* (Figure S5A-B). In soft agar and matrigel invasion assays, the anchorage-independent growth and invasion of SH-SY5Y and BE(2)-C cells were enhanced and reduced by stable ectopic expression or knockdown of *MZF1*, which was partially rescued by silencing or over-expression of *HK2* and *PGK1*, respectively (Figure S6A-B). In addition, treatment with glycolysis inhibitor (2-DG) 26 abolished the increase in glucose uptake, lactate production, ATP levels, growth and invasion of NB cells induced by stable *MZF1* over-expression (Figure S5B and Figure S6A-B). *In vivo* experiments using xenograft models revealed that stable over-expression of *MZF1* promoted the tumorigenecity of SH-SY5Y cells, as displayed by increase in tumor growth, tumor weight, Ki-67 proliferative index, and elevated levels of *HK2* and *PGK1* (Figure S7A). In contrast, stable silencing of *MZF1* into BE(2)-C cells decreased the growth, weight, Ki-67 proliferation index, and expression levels of *HK2* and *PGK1* of subcutaneous xenograft tumors in nude mice (Figure S7B).

In experimental metastasis assay, nude mice treated with tail vein injection of SH-SY5Y cells stably over-expressing *MZF1* presented more lung metastatic counts and lower survival possibility (Figure S7C), while stable knockdown of *MZF1* into BE(2)-C cells resulted in less lung metastatic colonies and greater survival probability in nude mice (Figure S7D). Moreover, administration of 2-DG prevented the increased tumorigenesis and aggressiveness of NB cells *in vivo* induced by stable *MZF1* over-expression (Figure S7A and Figure S7C). These results suggested that *MZF1* promoted tumor progression via facilitating aerobic glycolysis in NB.

### *MZF1-uORF*-encoded peptide inhibits *MZF1* expression

**Figure 3:**
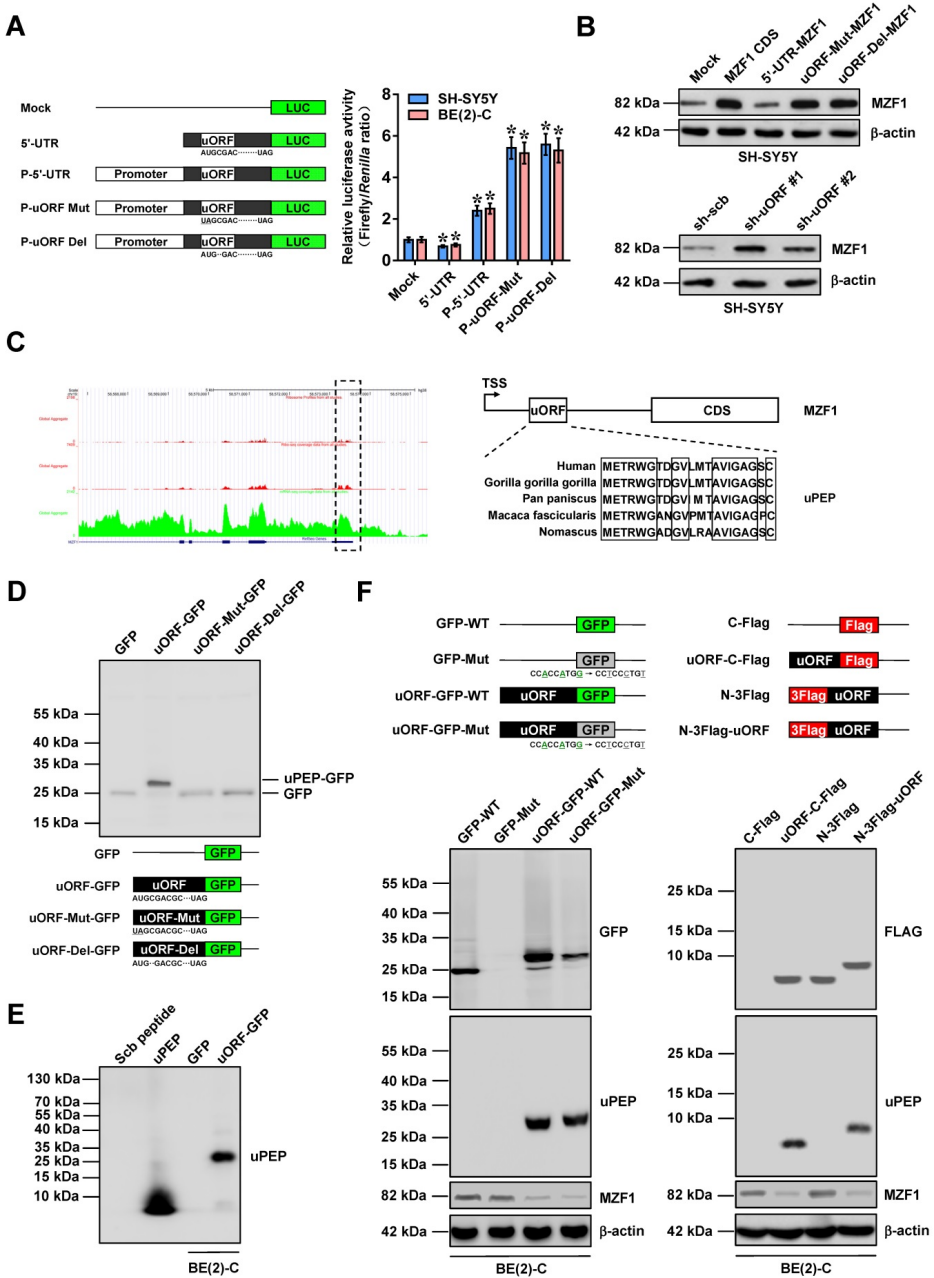
***MZF1-uORF*-encoded peptide inhibits *MZF1* expression. (A)** Dual-luciferase assay (right panel) indicating the activity of luciferase reporters containing wild-type, mutant, or deletion forms of *uORF* within 5'-UTR and promoter fragment of *MZF1* (left panel) in SH-SY5Y and BE(2)-C cells (*n*=4). **(B)** Western blot assay showing the levels of MZF1 in SH-SY5Y cells transfected with empty vector (mock), *MZF1* coding sequence (CDS), *MZF1* containing wild-type, mutant, or deletion forms of 5'-UTR, scramble shRNA (sh-Scb), or sh-uORF. **(C)** Mining of GWIPS-viz database (left panel) revealing the ribosome profiling at uORF region of *MZF1* (outlined), with homology of *MZF1-uORF*-encoded amino acid sequence as indicated (right panel). **(D)** Western blot assay using antibody specific for GFP (upper panel) indicating the expression of GFP and MZF1-uPEP-GFP fusion protein in HEK293 cells transfected with *GFP* or wild-type, mutation, or deletion forms of *MZF1-uORF-GFP* as indicated (lower panel). **(E)** Western blot assay using MZF1-uPEP specific antibody showing the expression of MZF1-uPEP and MZF1-uPEP-GFP fusion protein in BE(2)-C cells transfected with *GFP* or *MZF1-uORF-GFP*, with synthesized scramble (Scb) peptide or uPEP as controls. **(F)** Western blot assay (lower panel) indicating the expression of GFP, MZF1-uPEP, or MZF1 in BE(2)-C cells transfected with wild-type or mutant *GFP*, *MZF1-uORF-GFP* constructs, or Flag-tagged *MZF1-uORF* constructs as indicated (upper panel). ANOVA compared the difference in **A**. * *P*<0.05 vs. mock. Data are shown as mean ± s.e.m. (error bars) and representative of three independent experiments in **A**, **B**, and** D-F**.

To explore self-regulatory mechanisms underlying *MZF1* expression, we analyzed its 5'-UTR using ORF finder program (https://www.ncbi.nlm.nih.gov/orffinder), which revealed the existence of an uORF within this region (Figure 3A). Insertion of 5'-UTR of *MZF1* resulted in decrease of luciferase activity, while 5'-UTR along with *MZF1* promoter fragment facilitated the activity of luciferase reporter (Figure 3A). In addition, initiation codon mutation or frame-shift deletion of *uORF* within 5'-UTR led to significant increase in luciferase activity (Figure 3A). Transfection of *MZF1 CDS*, but not *MZF1 cDNA* containing 5'-UTR, resulted in increased protein and transcript levels of *MZF1* in SH-SY5Y cells (Figure 3B and Figure S8A). Initiation codon mutation or frame-shift deletion of *uORF* resulted in increase of *MZF1* levels in NB cells transfected by *MZF1 cDNA* containing 5'-UTR, while over-expression or knockdown of *uORF* led to decrease and increase in transcript and protein levels of *MZF1*, respectively (Figure 3B and Figure S8A-B). Further mining of SmPort 27 and GWIPS-viz 28 databases implicated that this ribosome-binding *uORF* might encode a 21-amino acid peptide with high conservation in primates (Figure 3C). Western blot assay using anti-GFP antibody indicated the fusion expression of uPEP with GFP in HEK293 cells, which was abolished by initiation codon mutation or frame-shift deletion of *uORF* (Figure 3D). The translation of uPEP-GFP protein in BE(2)-C cells was also validated by Coomassie blue staining and western blot using a rabbit polyclonal antibody against uPEP (Figure 3E and Figure S8C) and mutation of Kozak motif locating at upstream of GFP (Figure 3F). Ectopic expression of GFP-tagged or Flag-tagged uPEP resulted in obvious decrease of MZF1 levels (Figure 3F). Notably, in response to IGF1 stimulation, the distribution of *uORF* within heavy polysomes (within fractions 10-12) was decreased, while *MZF1* antisense RNA 1 (*MZF1-AS1*), a noncoding transcript containing complementary sequence of *uORF*, was not enriched in heavy polysomes (Figure S8D). Moreover, treatment with established glycolysis activator IGF1 29 led to phosphorylation of AKT, down-regulation of MZF1-uPEP, and up-regulation of MZF1 in BE(2)-C cells, which was abolished by phosphatidylinositol 3 kinase inhibitor LY294002 (Figure S8E). These data suggested that *MZF1-uORF*-encoded peptide inhibited *MZF1* expression at transcriptional level in NB cells.

### MZF1-uPEP interacts with YY1 to suppress its transactivation

**Figure 4:**
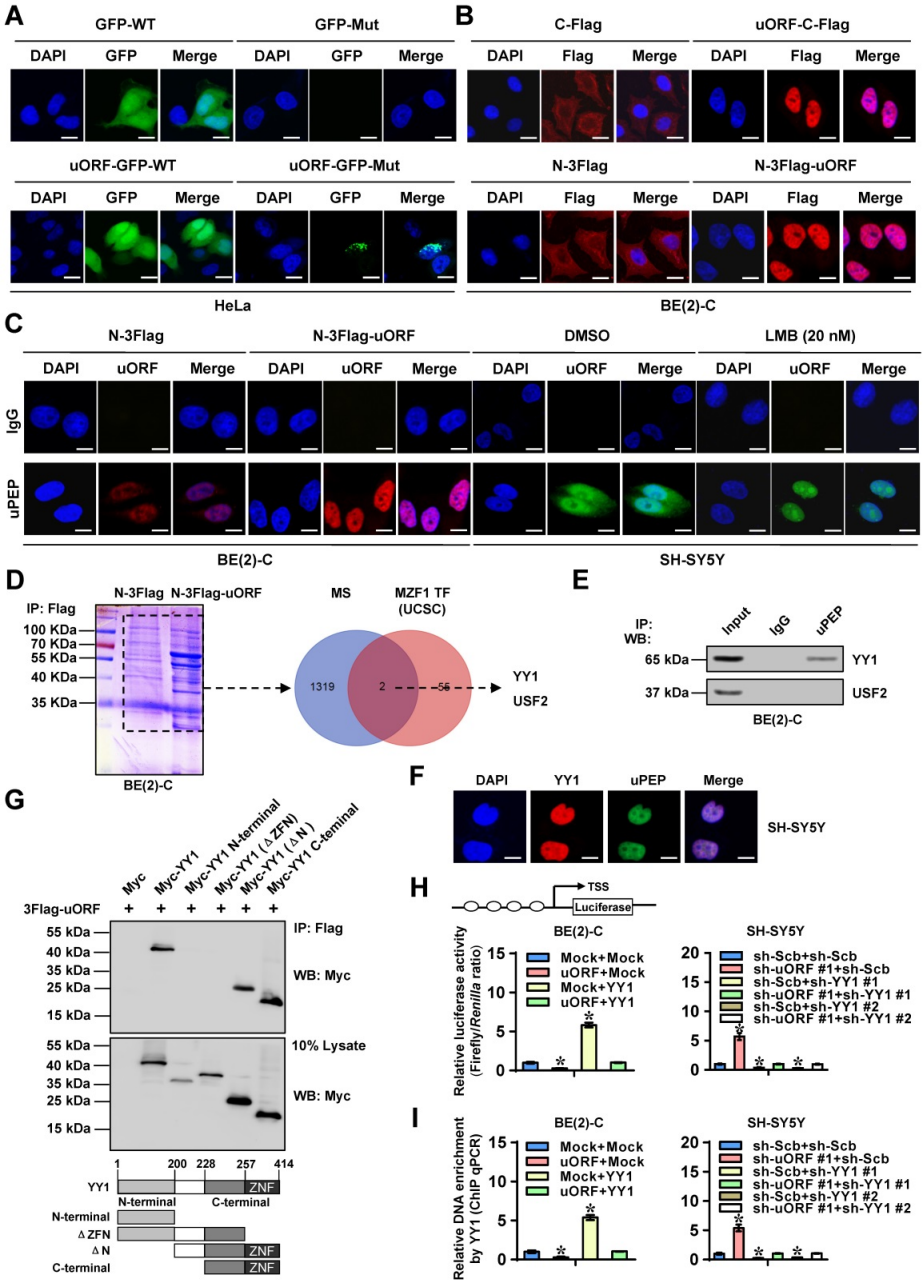
** MZF1-uPEP interacts with YY1 to suppress its transactivation. (A** and** B)** Confocal images showing the localization of MZF1-uPEP-GFP fusion protein or Flag-tagged MZF1-uPEP in HeLa and BE(2)-C cells transfected with wild-type (WT) or mutant (Mut) *GFP*, *MZF1-uORF-GFP* constructs, or Flag-tagged *MZF1-uORF*. **(C)** Immunofluorescence assay using MZF1-uPEP specific antibody indicating the localization of MZF1-uPEP in BE(2)-C cells transfected with N-terminal Flag vector or Flag-tagged *MZF1-uORF*, and that of SH-SY5Y cells treated with DMSO or LMB (20 nmol/L) for 48 hrs. **(D)** Coomassie blue staining (left panel) and Venn diagram (right panel) showing mass spectrometry (MS)-identified differential proteins pulled down by Flag antibody from BE(2)-C cells transfected with N-terminal Flag or Flag-tagged *MZF1-uORF*, and the over-lapping analysis with potential transcription factors of *MZF1* revealed by UCSC Genome Browser. **(E)** Co-IP and western blot assays revealing the interaction of MZF1-uPEP with YY1 or USF2 in BE(2)-C cells. **(F)** Immunofluorescence staining assay showing the co-localization of MZF1-uPEP (green) and YY1 (red) in SH-SY5Y cells, with nuclei stained by DAPI (blue). Scale bar: 10 μm. **(G)** Co-IP and western blot assays (upper panel) revealing the interaction between MZF1-uPEP and YY1 in BE(2)-C cells transfected with Flag-tagged *MZF1-uORF* and full-length or truncations of Myc-tagged *YY1* as indicated (lower panel). **(H** and** I)** Dual-luciferase (H) and ChIP qPCR (I) assays showing the activity of reporter containing four canonical YY1 binding sites and binding of YY1 to *MZF1* promoter in NB cells stably transfected with mock, *MZF1-uORF*, sh-Scb, or sh-uORF, and those co-transfected with *YY1* or sh-YY1 (*n*=4). ANOVA compared the difference in **H** and **I**. * *P*<0.05 vs. mock or sh-Scb. Data are shown as mean ± s.e.m. (error bars) and representative of three independent experiments in **A**-**C** and** E-I**.

To elucidate the mechanisms underlying MZF1-uPEP-inhibited *MZF1* expression, we first observed its subcellular localization. As shown in Figure 4A-B, GST-tagged or Flag-tagged MZF1-uPEP was mainly expressed within the nuclei of HeLa and BE(2)-C cells. Immunofluorescence assay using MZF1-uPEP specific antibody also revealed the nuclear or cytoplasmic enrichment of MZF1-uPEP in BE(2)-C cells, which was enhanced by transfection of *MZF1-uORF* (Figure 4C). Treatment with leptomycin B (LMB), an established nuclear export inhibitor 30, resulted in obvious aggregation of MZF1-uPEP within the nucleus of SH-SY5Y cells (Figure 4C). Then, to identify the protein partner of MZF1-uPEP, we performed the co-IP followed by a proteomic analysis of pulled down proteins in BE(2)-C cells. Mass spectrometry revealed 1321 differential proteins between empty vector (mock) and *MZF1-uORF* transfection groups (Table S5), and two of them (Figure 4D) were potential transcription factors regulating *MZF1* expression revealed by UCSC Genome Browser (Table S6). Further validating co-IP and western blot assays indicated that YY1 protein, but not USF2, was able to interact with MZF1-uPEP in BE(2)-C cells, which was abolished by IGF1 treatment (Figure 4E and Figure S8F). Co-localization of MZF1-uPEP and YY1 was observed in the nucleus of NB cells (Figure 4F). Deletion-mapping experiments indicated that zinc finger (ZNF) domain of YY1 (amino acids 258-414) was required for its binding to MZF1-uPEP (Figure 4G). Notably, the expression of YY1 and its interaction with MZF1-uPEP were higher in NB cell lines, than that in normal dorsal root ganglia (Figure S8G). Importantly, stable over-expression or knockdown of *MZF1-uORF* resulted in decreased and increased transactivation of YY1, which was prevented by ectopic expression or silencing of *YY1* in BE(2)-C and SH-SY5Y cells, respectively (Figure 4H).

In addition, transfection of *YY1* into BE(2)-C cells led to increase in *MZF1* promoter activity, while mutation of YY1 binding site or stable transfection of *MZF1-uORF* abolished these effects (Figure S8H). Ectopic expression or knockdown of *MZF1-uORF* decreased and increased the binding of YY1* to MZF1* promoter in NB cells, which was abolished by stable over-expression or knockdown of *YY1*, respectively (Figure 4I). Collectively, these results indicated that MZF1-uPEP interacted with YY1 to suppress its transactivation in NB cells.

### MZF1-uPEP exerts tumor suppressive roles by repressing YY1

To further investigate the functional roles of MZF1-uPEP, we performed rescue studies in NB cells. Stable ectopic expression of *MZF1-uORF* prevented the increase in glucose uptake, lactate production, ATP levels, growth, invasion, and metastasis of NB cells *in vitro* and *in vivo* induced by IGF1 stimulation (Figure S9A-D). Stable transfection of *MZF1-uORF* or sh-uORF #1 led to significantly decreased and increased expression of* MZF1* and downstream genes (*HK2* and *PGK1*) in BE(2)-C and SH-SY5Y cells (Figure S10A-B), which was abolished by ectopic expression or knockdown of *YY1*, respectively (Figure S10A-B). Meanwhile, ectopic expression or silencing of *YY1* abolished the decrease or increase in glucose uptake, lactate production, and ATP levels of NB cells induced by stable over-expression or knockdown of *MZF1-uORF* (Figure S10C). In MTT colorimetric, soft agar, and matrigel invasion assays, over-expression or silencing of *YY1* reversed the decrease or increase of viability, growth and invasiveness of NB cells induced by stable ectopic expression or knockdown of *MZF1-uORF*, respectively (Figure S10D-F). These data indicated that MZF1-uPEP exerted tumor suppressive roles by repressing YY1.

### Therapeutic efficiency of cell-penetrating MZF1-uPEP

**Figure 5:**
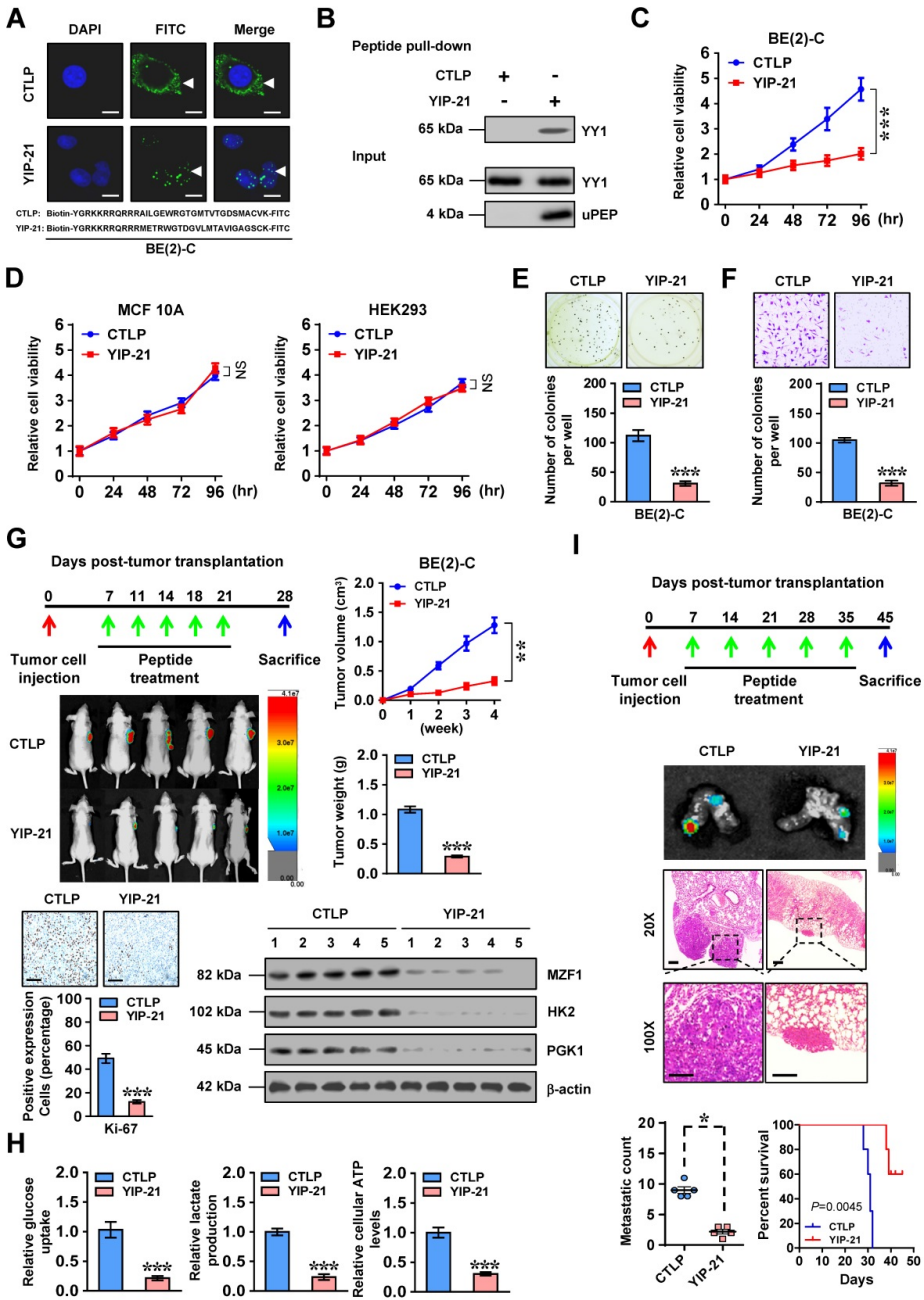
** Therapeutic efficiency of cell-penetrating MZF1-uPEP. (A)** Representative confocal images indicating the distribution of FITC-labeled control (CTLP) or YY1 inhibitory peptide (YIP-21, 20 μmol/L) in BE(2)-C cells, with nuclei stained by DAPI (blue). Scale bar: 10 μm. **(B)** Peptide pull-down assay showing the levels of YY1 pulled down by biotin-labeled CTLP or YIP-21 (20 μmol/L) from SH-SY5Y cells. **(C** and** D)** MTT colorimetric assay indicating the viability of BE(2)-C, MCF 10A, or HEK293 cells treated with CTLP or YIP-21 (20 μmol/L, *n*=6). **(E** and** F)** Representative images (upper panel) and quantification (lower panel) of soft agar (E) and matrigel invasion (F) assays showing the growth and invasion of BE(2)-C cells treated with CTLP or YIP-21 (20 μmol/L, *n*=4) for 48 hrs.** (G)** Representative images, *in vivo* growth curve, tumor weight, Ki-67 immunostaining, and expression of *MZF1* and downstream glycolytic genes within BE(2)-C-formed subcutaneous xenograft tumors (*n*=5 per group) in nude mice that treated with tail vein injection of CTLP or YIP-21 (3 mg·kg^-1^) as indicated. **(H)** Glucose uptake, lactate production, and ATP levels of BE(2)-C-formed subcutaneous xenograft tumors in nude mice (*n*=5 per group) that treated with tail vein injection of CTLP or YIP-21 (3 mg·kg^-1^). **(I)** Representative images (middle panels) and metastatic counts of lungs (lower left panel) and Kaplan-Meier curves (lower right panel) of nude mice (*n*=5 per group) treated with tail vein injection of BE(2)-C cells and CTLP or YIP-21 (3 mg·kg^-1^) as indicated (upper panel). Student's *t* test and ANOVA compared the difference in **C**-**I**. Log-rank test for survival comparison in **I**. * *P*<0.05, ** *P*<0.01, *** *P*<0.001 vs. CTLP. NS, non-significant. Data are shown as mean ± s.e.m. (error bars) and representative of three independent experiments in **A**-**F**.

Then, we further investigated the therapeutic efficiency of cell-penetrating MZF1-uPEP on biological behaviors of NB cells. Administration of a cell-penetrating FITC-labeled MZF1-uPEP with YY1 inhibiting properties, termed as YIP-21, resulted in its obvious nuclear enrichment in BE(2)-C cells (Figure 5A). Biotin-labeled peptide pull-down assay revealed that YIP-21, but not control peptide (CTLP), was able to bind with YY1 (Figure 5B). Administration of YIP-21 led to decrease in the viability of NB cells (Figure 5C), but not of non-transformed MCF 10A or transformed HEK293 cells without endogenous interaction between MZF1-uPEP and YY1 (Figure 5D and Figure S8G). In addition, treatment with YIP-21 decreased the anchorage-independent growth and invasion of BE(2)-C cells (Figure 5E-F). To test *in vivo* therapeutic potency of YIP-21, tail vein administration of YIP-21 or CTLP was performed in nude mice bearing subcutaneous xenograft tumors or lung metastasis formed by BE(2)-C cells. Administration of YIP-21 resulted in decreased growth and weight of subcutaneous xenograft tumors in nude mice (Figure 5G). The Ki-67-positive cells, expression of *MZF1* and its downstream genes, glucose uptake, lactate production, and ATP levels within subcutaneous xenograft tumors were also significantly reduced by YIP-21 treatment (Figure 5G-H). In experimental metastasis assay, nude mice treated with YIP-21 presented with less lung metastatic counts and longer survival time (Figure 5I). Consequently, these results demonstrated that cell-penetrating MZF1-uPEP suppressed tumorigenesis and aggressiveness of NB cells.

### Therapeutic efficiency of *MZF1-uORF* over-expression

To further assess the therapeutic efficacy of lentivirus-mediated *MZF1-uORF* over-expression on tumor progression, nude mice were treated with subcutaneous or tail vein injection of IMR-32 cells stably expressing red fluorescent protein. One week later, mice were randomly divided into groups, and received intravenous administration of lentivirus carrying empty vector (mock) or *MZF1-uORF*. Administration of lentivirus-mediated *MZF1-uORF* dramatically reduced the growth and weight of xenograft tumors (Figure S11A), decreased the Ki-67 proliferation index (Figure S11A), increased the MZF1-uPEP levels (Figure S11B), inhibited the expression of *MZF1* and downstream glycolytic genes (Figure S11C-D), and attenuated the glucose uptake, lactate production, and ATP levels within xenograft tumors (Figure S11E). In experimental metastasis assay, nude mice treated with tail vein administration of lentivirus-mediated *MZF1-uORF* presented fewer lung metastatic counts and longer survival time (Figure S11F). These data indicated that lentivirus-mediated over-expression of* MZF1-uORF* suppressed NB progression.

### MZF1-uPEP/*YY1*/*MZF1* expression is associated with NB outcome

**Figure 6:**
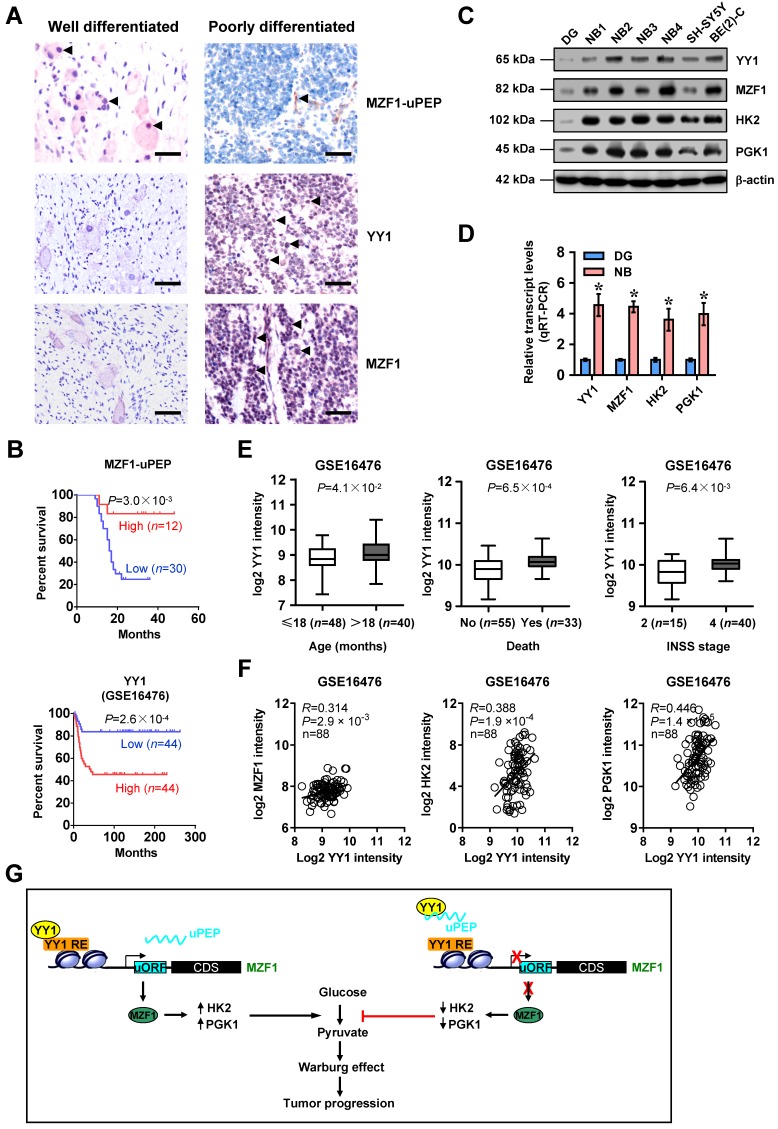
** MZF1-uPEP/*YY1*/*MZF1* expression is associated with NB outcome. (A)** Representative images of immunohistochemical staining showing the expression patterns of MZF1-uPEP, YY1, and MZF1 in tumor cells of NB specimens (arrowheads, brown). Scale bars: 50 μm. **(B)** Kaplan-Meier curves indicating overall survival of 42 NB patients with high or low MZF1-uPEP immunostaining in 88 (GSE16476) NB cases with low or high expression levels of *YY1* (cutoff value=1003.1). **(C** and** D)** Western blot (C) and real-time qRT-PCR (D, normalized to β-actin) assays showing the expression of *YY1*,* MZF1*, and target genes in normal dorsal root ganglia (DG), NB tissues (*n*=42), and NB cell lines. **(E)** Mining of a public microarray dataset (GSE16476) revealing the levels of *YY1* in NB tissues with different status of age, death, or INSS stages. **(F)** The positive expression correlation of* YY1* with *MZF1*, *HK2*, or *PGK1* in 88 NB cases (GSE16476). **(G)** The mechanisms underlying MZF1-uPEP-suppressed tumor progression: as an uORF-encoded small peptide, MZF1-uPEP directly binds to YY1 to repress its transactivation, resulting in decreased transcription of *MZF1* and downstream target glycolytic genes, and reduced aerobic glycolysis and tumor progression. Log-rank test for survival comparison in **B**. Student's *t* test compared the difference in **D** and** E**. Pearson's correlation coefficient analysis for gene expression in **F**. * *P*<0.05 vs. DG. Data are shown as mean ± s.e.m. (error bars) and representative of three independent experiments in **C** and **D**. Bars are means and whiskers (min to max) in **E**.

We then analyzed the significance of MZF1-uPEP, *YY1*, *MZF1*, and target genes in NB. Immunohistochemical staining revealed that MZF1-uPEP was expressed in nuclei and cytoplasm of tumor cells (Figure 6A and Figure S12A), and detected in 22/42 (52.4%) NB cases, with lower expression in those with elder age (*P*=0.038), poor differentiation (*P*=0.024), higher mitosis karyorrhexis index (MKI, *P*=0.037), or advanced *INSS* stages (*P*=0.007, Table S7). In these patients, low MZF1-uPEP expression was associated with poor survival probability (*P*=3.0×10^-3^, Figure 6B). Meanwhile, nuclear expression of YY1 (29/42) and MZF1 (26/42) was observed in these NB cases (Figure 6A and Table S8). The immunostaining of MZF1-uPEP and YY1 was negatively or positively associated with MZF1 immunoreactivity in NB cases, respectively (Table S9). Higher expression of *YY1*, *MZF1*, *HK2*, or *PGK1* was observed in NB tissues, than that in normal dorsal root ganglia (Figure 6C-D). High *YY1* expression was associated with poor outcome of patients with NB (GSE16476 and GSE62564, *P*=2.6×10^-4^ and *P*=4.9×10^-12^), breast cancer (*P*=3.1×10^-3^), endometrial carcinoma (*P*=6.1×10^-3^), glioma (*P*=1.6×10^-11^), head and neck carcinoma (*P*=1.2×10^-2^), lung cancer (*P*=5.5×10^-3^), lymphoma (*P*=1.0×10^-3^), pancreatic cancer (*P*=1.4×10^-4^), or renal clear cell carcinoma (*P*=3.0×10^-4^, Figure 6B and Figure S12B-C). In 88 NB cases (GSE16476), high levels of *YY1* were noted in tissues with elder age (*P*=4.1×10^-2^), death (*P*=6.5×10^-4^), or advanced INSS stage (*P*=6.4×10^-3^, Figure 6E). In addition, *YY1* expression was positively correlated with that of *MZF1*, *HK2*, or *PGK1* in these NB tissues (Figure 6F). These results indicated that expression of MZF1-uPEP/*YY1*/*MZF1* was associated with NB outcome.

## Discussion

Aerobic glycolysis facilitates malignant cell transformation, tumor initiation and aggressive progression 5, while inhibition of glycolysis impairs growth and metastasis of many tumor cells 10, 11, indicating an efficient therapeutic approach for tumors. Recent studies show that *LDHA* and *LDHB* are dispensable for aerobic glycolysis in NB 31, suggesting involvement of other glycolytic genes in this process. Among them, *HK2* is mainly expressed in cancers, and phosphorylates glucose to produce glucose-6-phosphate, a rate-limiting and irreversible step of glycolysis 8. In mouse models, *HK2* plays a vital role in tumor initiation and maintenance 32. Elevated *HK2* is associated with poor survival of hepatocellular carcinoma, while inhibition of *HK2* expression abrogates the tumorigenesis of tumor cells 33. PGK1, a rate-limiting enzyme of glycolytic pathway, catalyzes the transfer of high-energy phosphate from 1-position of 1,3-diphosphoglycerate to ADP, and is essential for ATP generation 34. *PGK1* is up-regulated in breast cancer 35, pancreatic ductal adenocarcinoma 36, and hepatocellular carcinoma 37, while depletion of *PGK1* dramatically reduces the proliferation and metastasis of cancer cells, indicating an oncogenic role of *PGK1* in tumor progression 38. In this study, we identify MZF1 as a transcription factor facilitating the expression of glycolytic genes *HK2* and *PGK1* in NB. In addition, we demonstrate that a peptide encoded by *MZF1-uORF* binds to YY1, resulting in decreased transactivation of YY1 and repressed expression of *MZF1* and downstream glycolytic genes *HK2* and *PGK1* in NB cells (Figure 6G), implying a negative feedback loop of uORF-encoded peptide in *MZF1* expression. Meanwhile, in response to stimulation of glycolysis activator, MZF1-uPEP is down-regulated to disrupt this negative feedback loop, resulting in enhanced MZF1 expression and aerobic glycolysis of NB cells.

*MZF1*, one member of Kruppel family proteins, is essential for the differentiation, proliferation, and migration of hematopoietic cells 39, 40. As a bi-functional transcription factor, MZF1 contains 13 zinc finger domains, and represses or activates gene transcription via binding to promoters 40. Recent studies show that *MZF1* plays an important role in tumorigenesis and aggressiveness. Forced expression of *MZF1* induces malignant transformation of NIH3T3 cells, and initiates tumor formation in athymic mice 41. MZF1 is involved in the etiology of many solid tumors, such as lung cancer 42, breast cancer 43, colorectal cancer 44, hepatocellular carcinoma 45, and cervical cancer 46. MZF1 facilitates the transcription of *c-MYC*, and is responsible for growth, migration, and invasion of lung adenocarcinoma cells 42. In breast cancer, MZF1 activates the expression of cathepsin B to increase the invasion of cancer cells 43. Over-expression of *MZF1* leads to transactivation of anexelekto (*AXL*) promoter and increase of migratory, invasive, and metastatic potential of colorectal cancer cells 44. In hepatocellular carcinoma, MZF1 enhances the transcription of protein kinase C alpha (*PKCα*), thus facilitating the migration and invasion of cancer cells 45. Meanwhile, MZF1 suppresses the migratory and invasive capability of cervical cancer cells by inhibiting transcription of matrix metalloproteinase-2 (*MMP-2*) 46. These results indicate that *MZF1* exerts oncogenic or tumor suppressive roles via transcriptional changes associated with malignant cell migration and invasiveness in a context-dependent manner. However, the roles of MZF1 in aerobic glycolysis during tumor progression still remain elusive. In this study, *MZF1* was identified as an independent prognostic factor for poor outcome of NB patients, while *HK2* and *PGK1* were direct target genes of *MZF1*. Our gain- and loss-of-function studies indicated that *MZF1* promoted aerobic glycolysis, growth, and invasiveness of NB cells, suggesting the oncogenic roles of *MZF1* in NB progression.

The widespread presence of uORF within 5'-UTR is one of the mechanisms regulating gene expression 47, 48. Approximate 50% of human transcripts contain uORF 49, and uORF is able to repress translation of mRNAs through disturbing ribosomal scanning or altering mRNA stability 50. For example, sex lethal protein binds to a *cis*-regulatory element within uORF, and imposes a negative effect on protein translation in *Drosophila* 51. Recent ribosome profiling and validating studies indicate the generation of short peptides encoded by uORFs 47, 50, and some uORF-encoded peptides are important for translational regulation 52, 53. The 5'-UTR of CCAAT/enhancer-binding protein homologous protein (*CHOP*) contains a conserved uORF which encodes a 31-amino acid peptide that inhibits the translation of *CHOP* 54. In this study, we identified a conserved uORF within *MZF1* 5'-UTR, which encoded a small peptide that bound to YY1 protein. Notably, MZF1-uPEP inhibited YY1-facilitated transcription of *MZF1*, indicting a novel action mode of uORF-encoded peptide in regulating gene transcription rather than protein translation. In addition, tumor suppressive functions of MZF1-uPEP were mediated, at least in part, through interacting with YY1 protein in NB cells.

*YY1* is a transcription factor of GLI-Kruppel family, and plays a regulatory role in cellular growth, oncogenic transformation, epithelial-mesenchymal transition, and metastasis 55. Human YY1 protein possesses a transactivation domain, a repression domain, and four C2H2-type zinc fingers 55, and activates or inactivates gene transcription depending on promoter contexts 56. *YY1* is highly expressed in many types of cancerous tissues, including prostate cancer, colon cancer, liver cancer, and lung cancer 57. In colon cancer, *YY1* promotes the growth and Wnt signaling pathway of cancer cells through inhibiting *p53* 58. In addition, *YY1* facilitates the transcription of p-glycoprotein in acute lymphoblastic lekeumia, and is associated with poor survival of patients 59. In this study, we found that *YY1* promoted the expression of *MZF1* in NB cells, resulting in facilitated glycolytic gene expression and tumor progression. In addition, MZF1-uPEP bound to zinc finger domain of YY1, resulting in repression of *YY1* transactivation in NB cells. Importantly, administration of a cell-penetrating MZF1-uPEP or lentivirus over-expressing MZF1-uPEP was able to suppress aerobic glycolysis, tumorigenesis, and aggressiveness of NB cells, suggesting the crucial roles of MZF1-uPEP in repressing *YY1*/*MZF1* axis in aerobic glycolysis and tumor progression.

## Conclusions

In summary, we demonstrate that *MZF1* is associated with poor outcome of NB, and exerts oncogenic roles in aerobic glycolysis and tumor progression. Meanwhile, *MZF1-*uORF-encoded peptide suppresses the *MZF1* expression, aerobic glycolysis, growth, and aggressiveness of NB cells. Mechanistically, *MZF1* promotes the expression of glycolytic genes *HK2* and *PGK1*, while MZF1-uPEP binds to YY1 to repress its transactivation, resulting in transcriptional suppression of *MZF1* and downstream glycolytic genes. Administration of a cell-penetrating MZF1-uPEP or lentivirus over-expressing MZF1-uPEP suppresses the aerobic glycolysis, tumorigenesis, and aggressiveness of NB cells. Since MZF1 expression is negatively regulated by microRNAs let-7e and let-7d in breast cancer cells 60, the roles of let-7 family members in regulating MZF1-mediated aerobic glycolysis during NB progression warrant investigation. In addition, further studies are needed to explore the potential roles of noncoding RNA *MZF1-AS1* in regulating MZF1-uPEP expression in NB. We believe that this study extends our knowledge about the regulation of aerobic glycolysis by transcription factor and its derived uPEP, and suggests that *MZF1* and *YY1* may be potential therapeutic targets for tumor progression.

## Supplementary Material

Supplementary figures and tables.

## Abbreviations

Co-IP: co-immunoprecipitation
ECAR: extracellular acidification rate
HK2: hexokinase 2
MZF1: myeloid zinc finger 1
NB: neuroblastoma
OCR: oxygen consumption rate
PGK1: phosphoglycerate kinase 1
qPCR: quantitative PCR
qRT-PCR: quantitative RT-PCR
sgRNA: single guide RNA
shRNA: short hairpin RNA
YY1: Yin Yang 1

## References

[1] Maris J (2010). Recent advances in neuroblastoma. N Engl J Med.

[2] Zhang Q, Zhang Q, Jiang X, Ye Y, Liao H, Zhu F (2019). Collaborative ISL1/GATA3 interaction in controlling neuroblastoma oncogenic pathways overlapping with but distinct from MYCN. Theranostics.

[3] Villasante A, Sakaguchi K, Kim J, Cheung NK, Nakayama M, Parsa H (2017). Vascularized tissue-engineered model for studying drug resistance in neuroblastoma. Theranostics.

[4] Warburg O (1956). On the origin of cancer cells. Science.

[5] Hanahan D, Weinberg RA (2011). Hallmarks of cancer: the next generation. Cell.

[6] Chen M, Sheng XJ, Qin YY, Zhu S, Wu QX, Jia L (2019). TBC1D8 amplification drives tumorigenesis through metabolism reprogramming in ovarian cancer. Theranostics.

[7] Zheng YL, Li L, Jia YX, Zhang BZ, Li JC, Zhu YH (2019). LINC01554-mediated glucose metabolism reprogramming suppresses tumorigenicity in hepatocellular carcinoma via downregulating PKM2 expression and inhibiting Akt/mTOR signaling pathway. Theranostics.

[8] Mathupala SP, Ko YH, Pedersen PL (2009). Hexokinase-2 bound to mitochondria: Cancer's stygian link to the “Warburg effect” and a pivotal target for effective therapy. Semin Cancer Biol.

[9] Altenberg B, Greulich K (2004). Genes of glycolysis are ubiquitously overexpressed in 24 cancer classes. Genomics.

[10] Nilsson H, Lindgren D, Mandahl Forsberg A, Mulder H, Axelson H, Johansson ME (2015). Primary clear cell renal carcinoma cells display minimal mitochondrial respiratory capacity resulting in pronounced sensitivity to glycolytic inhibition by 3-Bromopyruvate. Cell Death Dis.

[11] Ciavardelli D, Rossi C, Barcaroli D, Volpe S, Consalvo A, Zucchelli M (2014). Breast cancer stem cells rely on fermentative glycolysis and are sensitive to 2-deoxyglucose treatment. Cell Death Dis.

[12] Yang F, Zhang H, Mei Y, Wu M (2014). Reciprocal regulation of HIF-1alpha and lincRNA-p21 modulates the Warburg effect. Mol Cell.

[13] Kim JW, Gao P, Liu YC, Semenza GL, Dang CV (2007). Hypoxia-inducible factor 1 and dysregulated c-Myc cooperatively induce vascular endothelial growth factor and metabolic switches hexokinase 2 and pyruvate dehydrogenase kinase 1. Mol Cell Biol.

[14] Shim H, Dolde C, Lewis BC, Wu CS, Dang G, Jungmann RA (1997). c-Myc transactivation of LDH-A: implications for tumor metabolism and growth. Proc Natl Acad Sci USA.

[15] Schwartzenberg-Bar-Yoseph F, Armoni M, Karnieli E (2004). The tumor suppressor p53 down-regulates glucose transporters GLUT1 and GLUT4 gene expression. Cancer Res.

[16] Zhao X, Li D, Pu J, Mei H, Yang D, Xiang X (2016). CTCF cooperates with noncoding RNA MYCNOS to promote neuroblastoma progression through facilitating MYCN expression. Oncogene.

[17] Li D, Wang X, Mei H, Fang E, Ye L, Song H (2018). Long noncoding RNA pancEts-1 promotes neuroblastoma progression through hnRNPK-mediated beta-catenin stabilization. Cancer Res.

[18] Li D, Song H, Mei H, Fang E, Wang X, Yang F (2018). Armadillo repeat containing 12 promotes neuroblastoma progression through interaction with retinoblastoma binding protein 4. Nat Commun.

[19] Zhao X, Li D, Huang D, Song H, Mei H, Fang E (2018). Risk-associated long noncoding RNA FOXD3-AS1 inhibits neuroblastoma progression by repressing PARP1-mediated activation of CTCF. Mol Ther.

[20] Jiao W, Chen Y, Song H, Li D, Mei H, Yang F (2018). HPSE enhancer RNA promotes cancer progression through driving chromatin looping and regulating hnRNPU/p300/EGR1/HPSE axis. Oncogene.

[21] Ma X, Li C, Sun L, Huang D, Li T, He X (2014). Lin28/let-7 axis regulates aerobic glycolysis and cancer progression via PDK1. Nat Commun.

[22] Ferrick D, Neilson A, Beeson C (2008). Advances in measuring cellular bioenergetics using extracellular flux. Drug Discov Today.

[23] Hall DM, Brooks SA (2014). *In vitro* invasion assay using matrigel™: a reconstituted basement membrane preparation. Methods Mol Biol.

[24] Molenaar JJ, Koster J, Zwijnenburg DA, van Sluis P, Valentijn LJ, van der Ploeg I (2012). Sequencing of neuroblastoma identifies chromothripsis and defects in neuritogenesis genes. Nature.

[25] Gilbert LA, Horlbeck MA, Adamson B, Villalta JE, Chen Y, Whitehead EH (2014). Genome-scale CRISPR-mediated control of gene repression and activation. Cell.

[26] Ciavardelli D, Rossi C, Barcaroli D, Volpe S, Consalvo A, Zucchelli M (2014). Breast cancer stem cells rely on fermentative glycolysis and are sensitive to 2-deoxyglucose treatment. Cell Death Dis.

[27] Hao Y, Zhang L, Niu Y, Cai T, Luo J, He S (2018). SmProt: a database of small proteins encoded by annotated coding and non-coding RNA loci. Brief Bioinform.

[28] Michel AM, Fox G, M Kiran A, De Bo C, O'Connor PB, Heaphy SM (2014). GWIPS-viz: development of a ribo-seq genome browser. Nucleic Acids Res.

[29] Salani B, Ravera S, Amaro A, Salis A, Passalacqua M, Millo E (2015). IGF1 regulates PKM2 function through Akt phosphorylation. Cell Cycle.

[30] Kudo N, Wolff B, Sekimoto T, Schreiner E, Yoneda Y, Yanagida M (1998). Leptomycin B inhibition of signal-mediated nuclear export by direct binding to CRM1. Exp Cell Res.

[31] Dorneburg C, Fischer M, Barth TFE, Mueller-Klieser W, Hero B, Gecht J (2018). LDHA in neuroblastoma is associated with poor outcome and its depletion decreases neuroblastoma growth independent of aerobic glycolysis. Clin Cancer Res.

[32] Patra K, Wang Q, Bhaskar P, Miller L, Wang Z, Wheaton W (2013). Hexokinase 2 is required for tumor initiation and maintenance and its systemic deletion is therapeutic in mouse models of cancer. Cancer Cell.

[33] DeWaal D, Nogueira V, Terry A, Patra K, Jeon S, Guzman G (2018). Hexokinase-2 depletion inhibits glycolysis and induces oxidative phosphorylation in hepatocellular carcinoma and sensitizes to metformin. Nat Commun.

[34] Bernstein B, Hol W (1998). Crystal structures of substrates and products bound to the phosphoglycerate kinase active site reveal the catalytic mechanism. Biochemistry.

[35] Zhang D, Tai L, Wong L, Chiu L, Sethi S, Koay E (2005). Proteomic study reveals that proteins involved in metabolic and detoxification pathways are highly expressed in HER-2/neu-positive breast cancer. Mol Cell Proteomics.

[36] Hwang T, Liang Y, Chien K, Yu J (2006). Overexpression and elevated serum levels of phosphoglycerate kinase 1 in pancreatic ductal adenocarcinoma. Proteomics.

[37] Ai J, Huang H, Lv X, Tang Z, Chen M, Chen T (2011). FLNA and PGK1 are two potential markers for progression in hepatocellular carcinoma. Cell Physiol Biochem.

[38] Xie H, Tong G, Zhang Y, Liang S, Tang K, Yang Q (2017). PGK1 Drives hepatocellular carcinoma metastasis by enhancing metabolic process. Int J Mol Sci.

[39] Hui P, Guo X, Bradford P (1995). Isolation and functional characterization of the human gene encoding the myeloid zinc finger protein MZF-1. Biochemistry.

[40] Eguchi T, Prince T, Wegiel B, Calderwood SK (2015). Role and regulation of myeloid zinc finger protein 1 in cancer. J Cell Biochem.

[41] Hromas R, Morris J, Cornetta K, Berebitsky D, Davidson A, Sha M (1995). Aberrant expression of the myeloid zinc finger gene, MZF-1, is oncogenic. Cancer Res.

[42] Tsai LH, Wu JY, Cheng YW, Chen CY, Sheu GT, Wu TC (2015). The MZF1/c-MYC axis mediates lung adenocarcinoma progression caused by wild-type lkb1 loss. Oncogene.

[43] Rafn B, Nielsen Christian F, Andersen Sofie H, Szyniarowski P, Corcelle-Termeau E, Valo E (2012). ErbB2-driven breast cancer cell invasion depends on a complex signaling network activating myeloid zinc finger-1-dependent Cathepsin B expression. Mol Cell.

[44] Mudduluru G, Vajkoczy P, Allgayer H (2010). Myeloid zinc finger 1 induces migration, invasion, and *in vivo* metastasis through Axl gene expression in solid cancer. Mol Cancer Res.

[45] Hsieh YH, Wu TT, Tsai JH, Huang CY, Hsieh YS, Liu JY (2006). PKCalpha expression regulated by Elk-1 and MZF-1 in human HCC cells. Biochem Biophys Res Commun.

[46] Tsai SJ, Hwang JM, Hsieh SC, Ying TH, Hsieh YH (2012). Overexpression of myeloid zinc finger 1 suppresses matrix metalloproteinase-2 expression and reduces invasiveness of SiHa human cervical cancer cells. Biochem Biophys Res Commun.

[47] Sachs MS, Geballe AP (2006). Downstream control of upstream open reading frames. Genes Dev.

[48] Zhang Y, Zhao T, Li W, Vore M (2010). The 5'-untranslated region of multidrug resistance associated protein 2 (MRP2; ABCC2) regulates downstream open reading frame expression through translational regulation. Mol Pharmacol.

[49] Calvo SE, Pagliarini DJ, Mootha VK (2009). Upstream open reading frames cause widespread reduction of protein expression and are polymorphic among humans. Proc Natl Acad Sci USA.

[50] Morris DR, Geballe AP (2000). Upstream open reading frames as regulators of mRNA translation. Mol Cell Biol.

[51] Medenbach J, Seiler M, Hentze MW (2011). Translational control via protein-regulated upstream open reading frames. Cell.

[52] Spevak CC, Ivanov IP, Sachs MS (2010). Sequence requirements for ribosome stalling by the arginine attenuator peptide. J Biol Chem.

[53] Wei J, Wu C, Sachs MS (2012). The arginine attenuator peptide interferes with the ribosome peptidyl transferase center. Mol Cell Biol.

[54] Jousse C, Bruhat A, Carraro V, Urano F, Ferrara M, Ron D (2001). Inhibition of CHOP translation by a peptide encoded by an open reading frame localized in the chop 5'UTR. Nucleic Acids Res.

[55] Gordon S, Akopyan G, Garban H, Bonavida B (2005). Transcription factor YY1: structure, function, and therapeutic implications in cancer biology. Oncogene.

[56] Thomas MJ, Seto E (1999). Unlocking the mechanisms of transcription factor YY1: are chromatin modifying enzymes the key?. Gene.

[57] Zaravinos A, Spandidos DA (2010). Yin Yang 1 expression in human tumors. Cell cycle.

[58] Zhang N, Li X, Wu CW, Dong Y, Cai M, Mok MT (2013). microRNA-7 is a novel inhibitor of YY1 contributing to colorectal tumorigenesis. Oncogene.

[59] Antonio-Andres G, Rangel-Santiago J, Tirado-Rodriguez B, Martinez-Ruiz GU, Klunder-Klunder M, Vega MI (2018). Role of Yin Yang-1 (YY1) in the transcription regulation of the multi-drug resistance (MDR1) gene. Leuk Lymphoma.

[60] Tvingsholm SA, Hansen MB, Clemmensen KKB, Brix DM, Rafn B, Frankel LB (2018). Let-7 microRNA controls invasion-promoting lysosomal changes via the oncogenic transcription factor myeloid zinc finger-1. Oncogenesis.

