# Supplementary material for: Therapeutic targeting of YY1/MZF1 axis by MZF1-uPEP inhibits aerobic glycolysis and neuroblastoma progression
Source: Theranostics. 2020 Jan 1;10(4):1555–71. doi: 10.7150/thno.37383 (PMC6993229; doi:10.7150/thno.37383)
Supplement: Supplementary file 1 — Supplementary figures and tables. [file thnov10p1555s1.pdf]

Supplementary Information

Therapeutic targeting of YY1/MZF1 axis by MZF1-uPEP inhibits aerobic glycolysis and neuroblastoma progression

Fang et al.

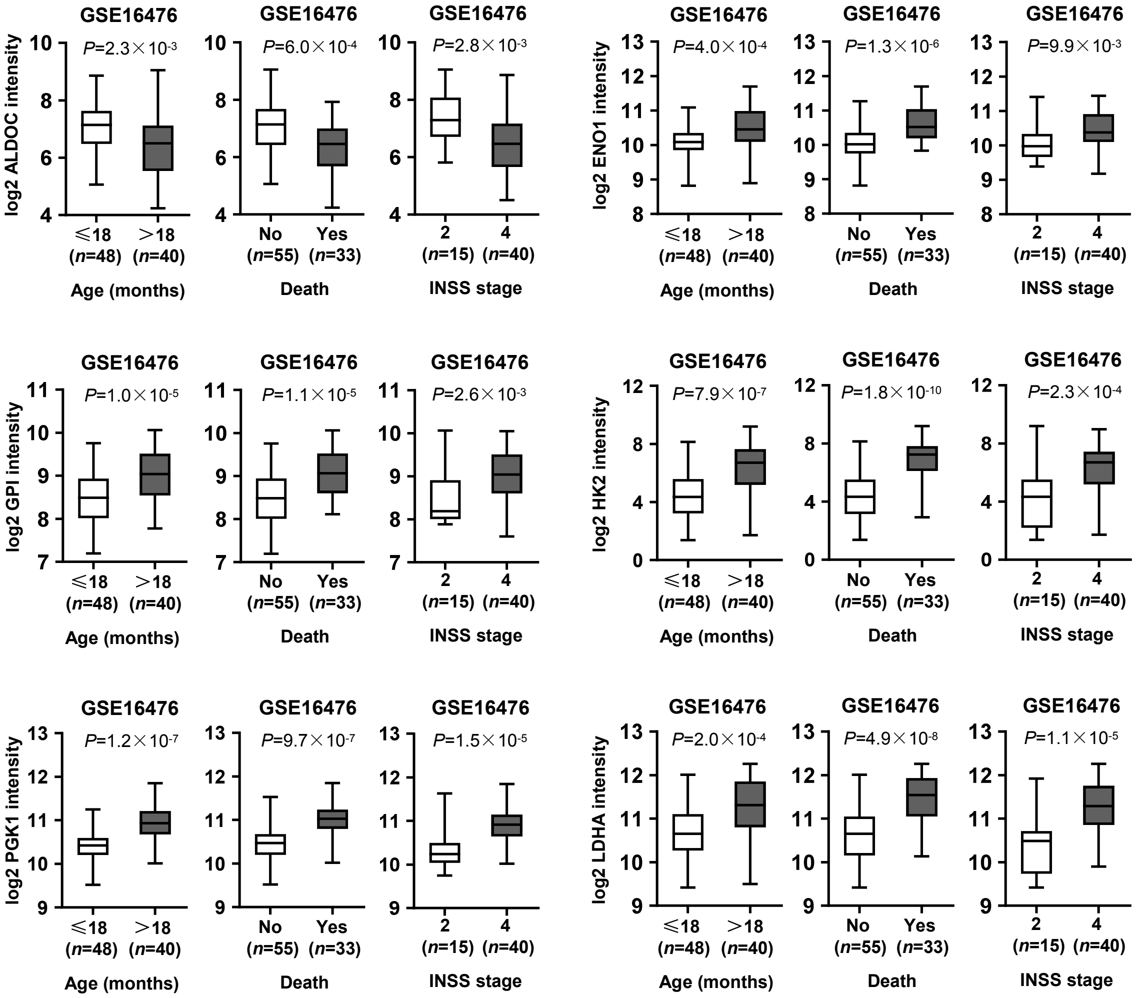

Figure S1. Expression of glycolytic genes in a public NB dataset. Mining of a public microarray dataset (GSE16476) revealing the levels of *ALDOC*, *ENO1*, *GPI*, *HK2*, *PGK1*, or *LDHA* in NB tissues with different status of age, death, or INSS stages. Student's *t* test compared the difference. Bars are means and whiskers (min to max).

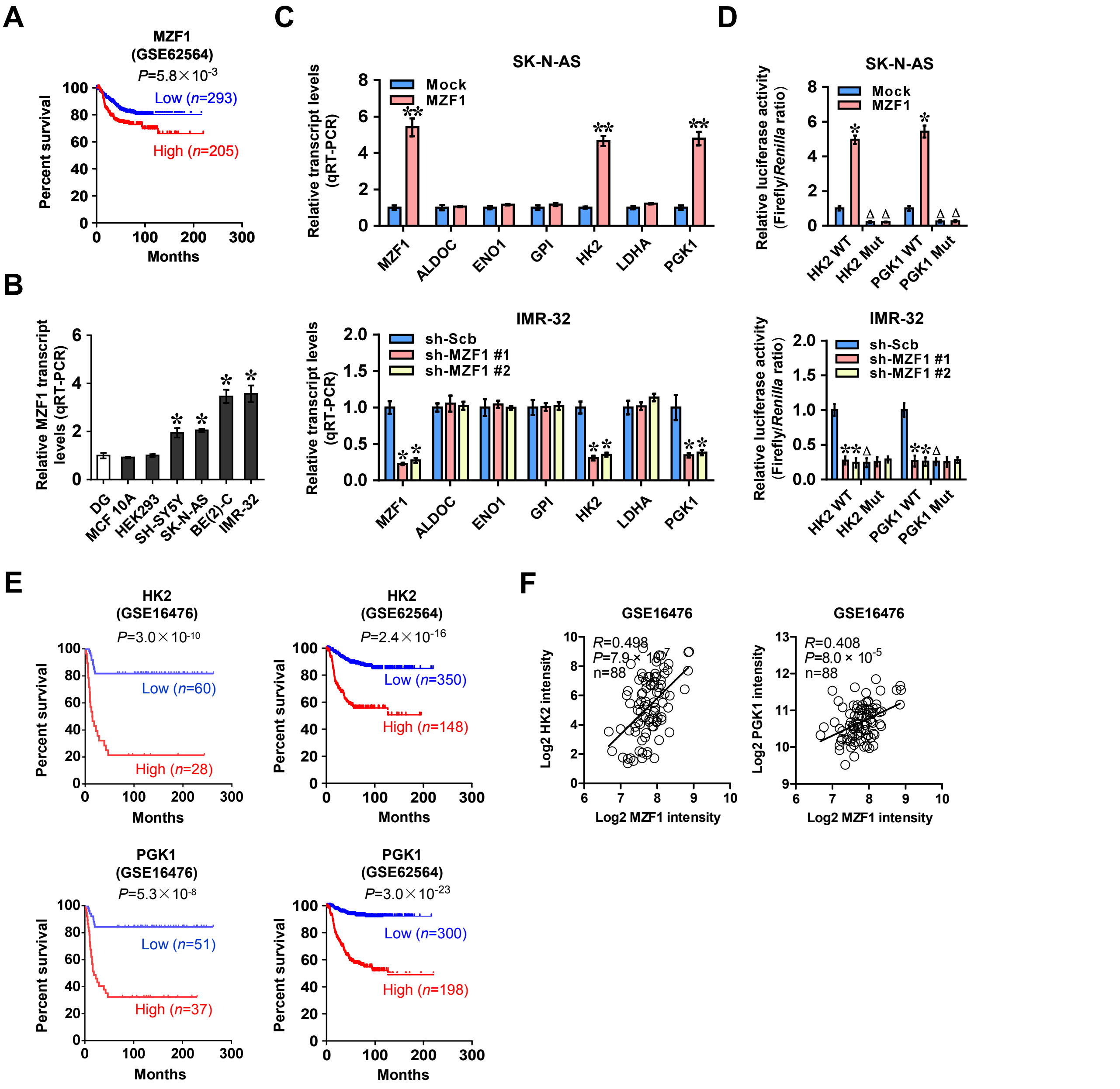

Figure S2. Roles of *MZF1* and downstream glycolytic genes in NB. (A) Kaplan-Meier curves indicating the overall survival of 498 NB cases (GSE62564) with low or high levels of *MZF1* (cutoff value=35.1). **(B)** Real-time qRT-PCR assay showing the transcript levels of *MZF1* in normal dorsal root ganglia (DG), non-transformed MCF 10A cells, transformed normal HEK293 cells, and NB cell lines (normalized to β-actin, *n*=4). **(C)** Real-time qRT-PCR assay (normalized to β-actin, *n*=4) indicating the transcript levels of *MZF1*, *ALDOC*, *ENO1*, *GPI*, *HK2*, *LDHA*, and *PGK1* in SK-N-AS and IMR-32 cells stably transfected with empty vector (mock), *MZF1*, scramble shRNA (sh-Scb), or sh-MZF1. **(D)** Dual-luciferase assay showing the promoter activity of *HK2* and *PGK1* with wild-type (WT) or mutant (Mut) MZF1 binding site in SK-N-AS and IMR-32 cells stably transfected with mock, *MZF1*, sh-Scb, or sh-MZF1 (*n*=6). **(E)** Kaplan-Meier curves indicating the overall survival of 88 (GSE16476) and 498 (GSE62564) NB cases with low or high levels of *HK2* (cutoff values=90.6 and 20.4) or *PGK1* (cutoff values=1735.5 and 98.6). **(F)** The expression correlation of *MZF1* with *HK2* or *PGK1* in 88 NB cases (GSE16476). Log-rank test for survival comparison in **A** and **E**. ANOVA and Student's *t* test compared the difference in **B-D**. Pearson's correlation coefficient analysis for gene expression in **F**. * P<0.05, ** P<0.01 vs. DG, mock, or sh-Scb. $^{\Delta}$ P<0.05 vs. WT. Data are shown as mean ± s.e.m. (error bars) and representative of three independent experiments in **B-D**.

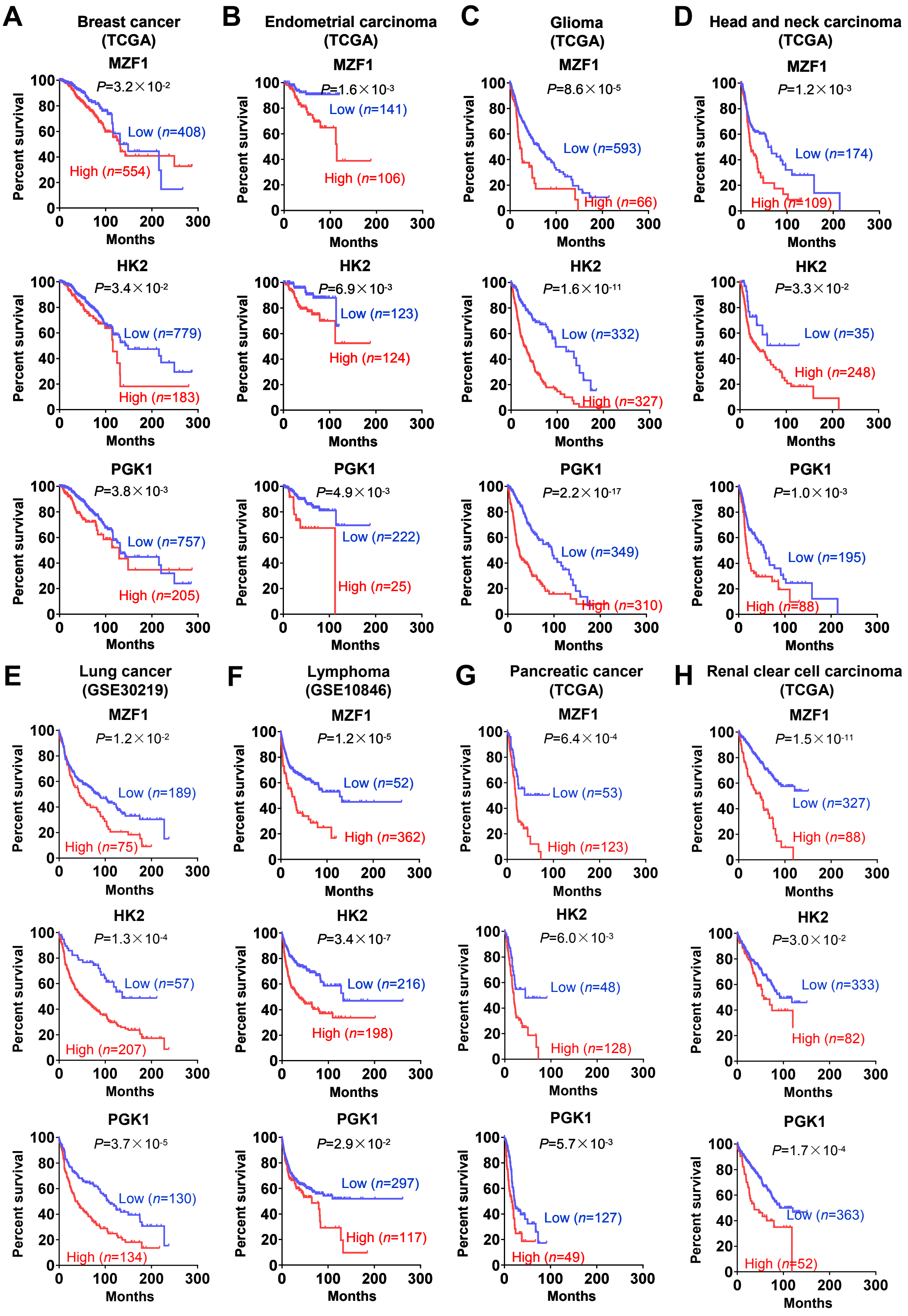

Figure S3. Kaplan–Meier survival plots of *MZF1*, *HK2*, and *PGK1* in public tumor datasets. Kaplan–Meier survival plots of patients with low or high levels of *MZF1*, *HK2*, and *PGK1* in TCGA or GEO datasets of breast cancer **(A)**, endometrial carcinoma **(B)**, glioma **(C)**, head and neck carcinoma **(D)**, lung cancer **(E)**, lymphoma **(F)**, pancreatic cancer **(G)**, or renal clear cell carcinoma **(H)**. Log-rank test for survival comparison.

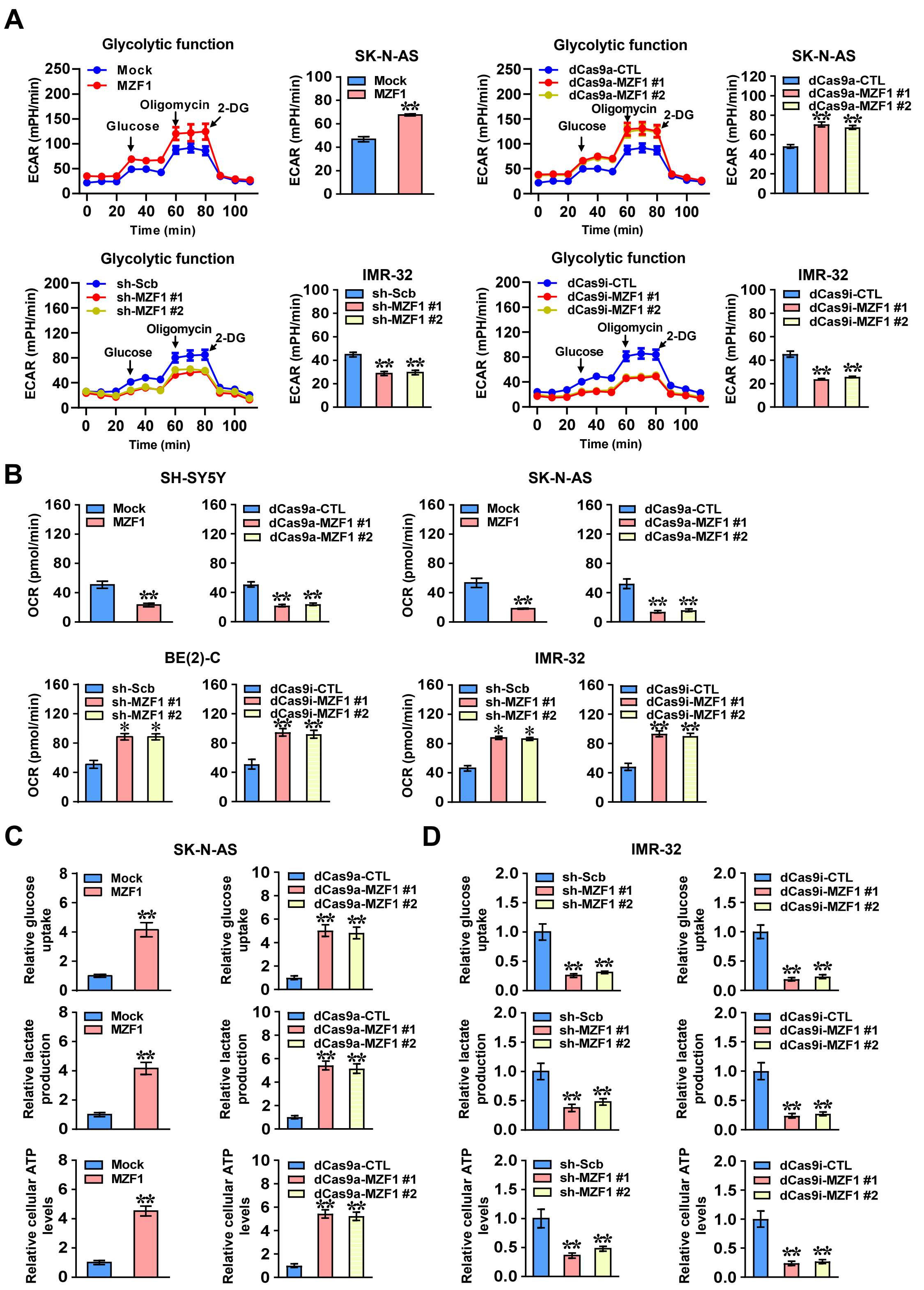

Figure S4. ***MZF1* promotes aerobic glycolysis of NB cells. (A)** Seahorse tracing curves (left panel) and ECAR bars (right panel) of SK-N-AS and IMR-32 cells stably transfected with empty vector (mock), *MZF1*, scramble shRNA (sh-Scb), sh-MZF1, dCas9a control vector (dCas9a-CTL), dCas9a-MZF1, dCas9i control vector (dCas9i-CTL), or dCas9i-MZF1, and those treated with glucose (10 mmol·L^{-1}), oligomycin (2 μmol·L^{-1}), or 2-deoxyglucose (2-DG, 100 mmol·L^{-1}) at indicated (4 replicates for each point). **(B)** Seahorse extracellular flux assay indicating the oxygen consumption rate (OCR) in SK-N-AS and IMR-32 cells stably transfected with mock, *MZF1*, sh-Scb, sh-MZF1, dCas9a-CTL, dCas9a-MZF1, dCas9i-CTL, or dCas9i-MZF1 (n=4). **(C** and **D)** Glucose uptake, lactate production, and ATP levels in NB cells stably transfected with mock, *MZF1*, sh-Scb, sh-MZF1, dCas9a-CTL, dCas9a-MZF1, dCas9i-CTL, or dCas9i-MZF1 (n=4). Student's t test and ANOVA compared the difference in **A**-**D**. * P<0.05, ** P<0.01 vs. mock, sh-Scb, dCas9a-CTL, or dCas9i-CTL. Data are shown as mean ± s.e.m. (error bars) and representative of three independent experiments in **A**-**D**.

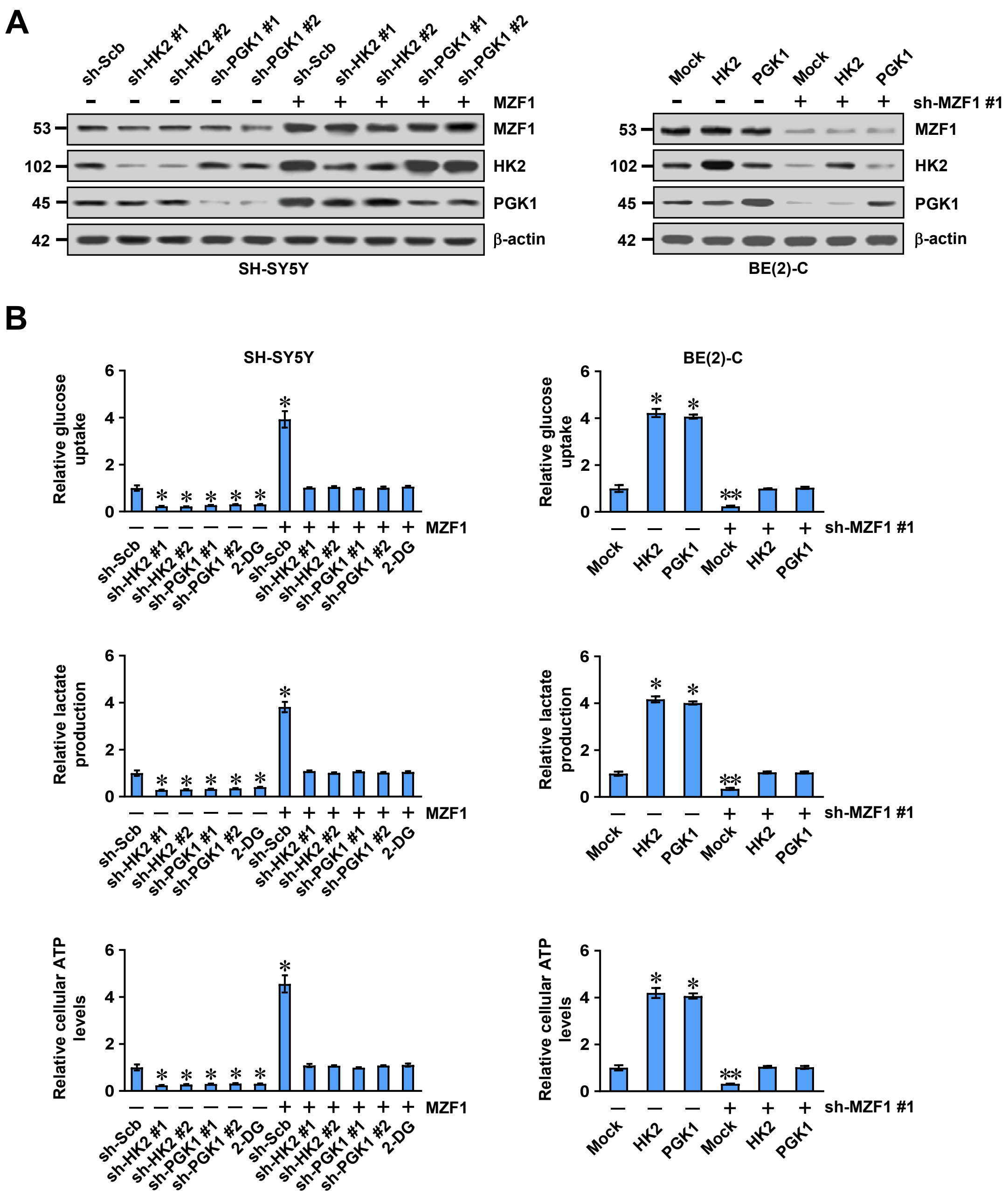

Figure S5. ***MZF1* promotes aerobic glycolysis of NB cells via up-regulating *HK2* and *PGK1*. (A)** Western blot assay indicating the expression of MZF1, HK2, and PGK1 in SH-SY5Y and BE(2)-C cells stably transfected with empty vector (mock), *MZF1*, scramble shRNA (sh-Scb), or sh-MZF1, and those co-transfected with sh-HK2, sh-PGK1, *HK2*, or *PGK1*. **(B)** Glucose uptake, lactate production, and ATP levels in SH-SY5Y and BE(2)-C cells stably transfected with mock, *MZF1*, sh-Scb, or sh-MZF1, and those co-transfected with sh-HK2, sh-PGK1, *HK2*, and *PGK1*, or treated with 2-DG (10 mmol·L^{-1}) for 48 hrs. ANOVA compared the difference in **B**. * $P<0.05$, ** $P<0.01$ vs. mock+sh-Scb. Data are shown as mean ± s.e.m. (error bars) and representative of three independent experiments in **A** and **B**.

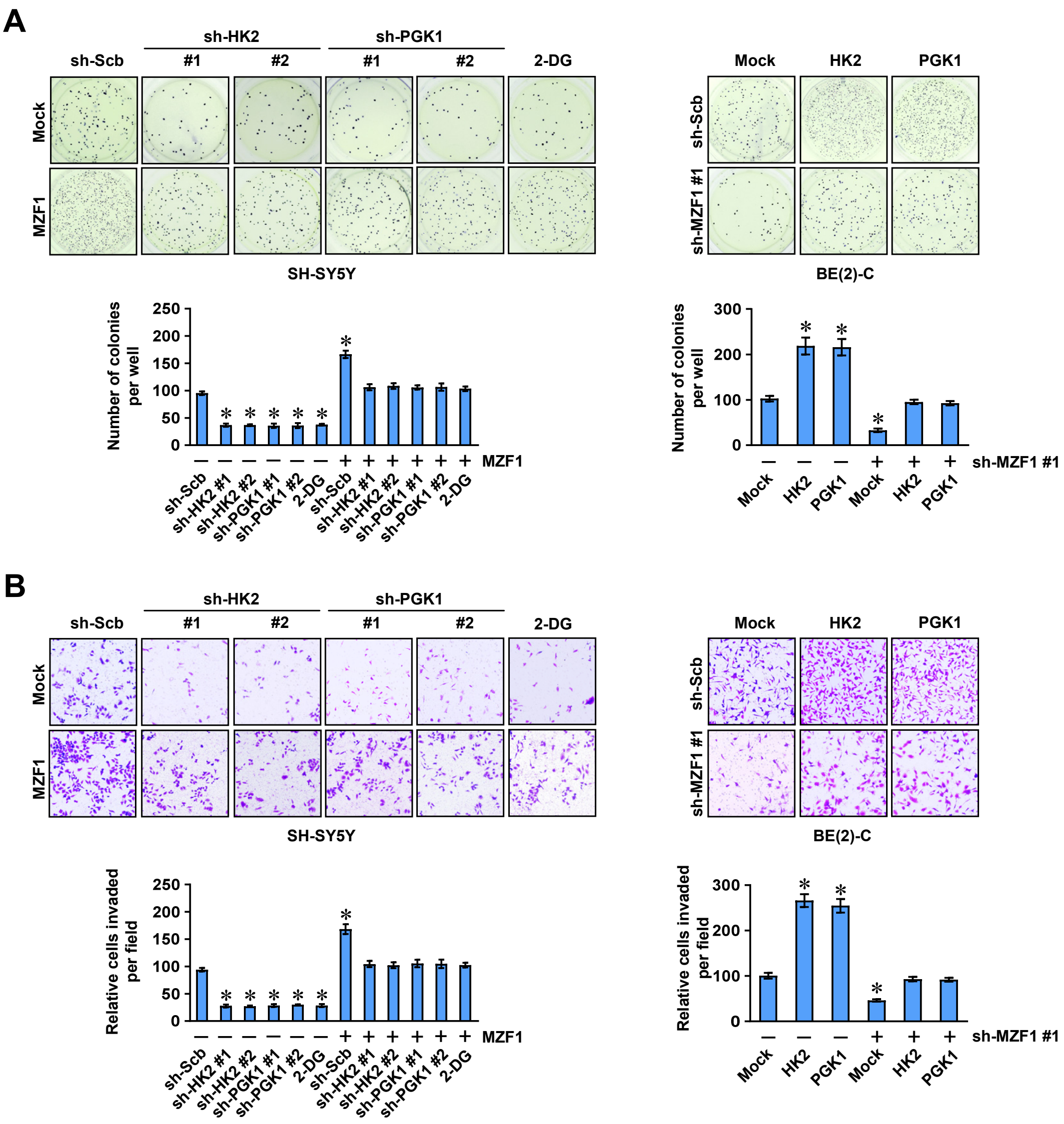

Figure S6. ***MZF1* promotes growth and invasion of NB cells via up-regulating *HK2* and *PGK1*.** Representative images (upper panel) and quantification (lower panel) of soft agar **(A)** and matrigel invasion **(B)** assays showing the growth and invasion of SH-SY5Y and BE(2)-C cells stably transfected with empty vector (mock), *MZF1*, scramble shRNA (sh-Scb), or sh-MZF1, and those co-transfected with sh-HK2, sh-PGK1, *HK2*, and *PGK1*, or treated with 2-DG (10 mmol·L^{-1}) for 48 hrs. ANOVA compared the difference in **A** and **B**. * P<0.05 vs. mock+sh-Scb. Data are shown as mean ± s.e.m. (error bars) and representative of three independent experiments in **A** and **B**.

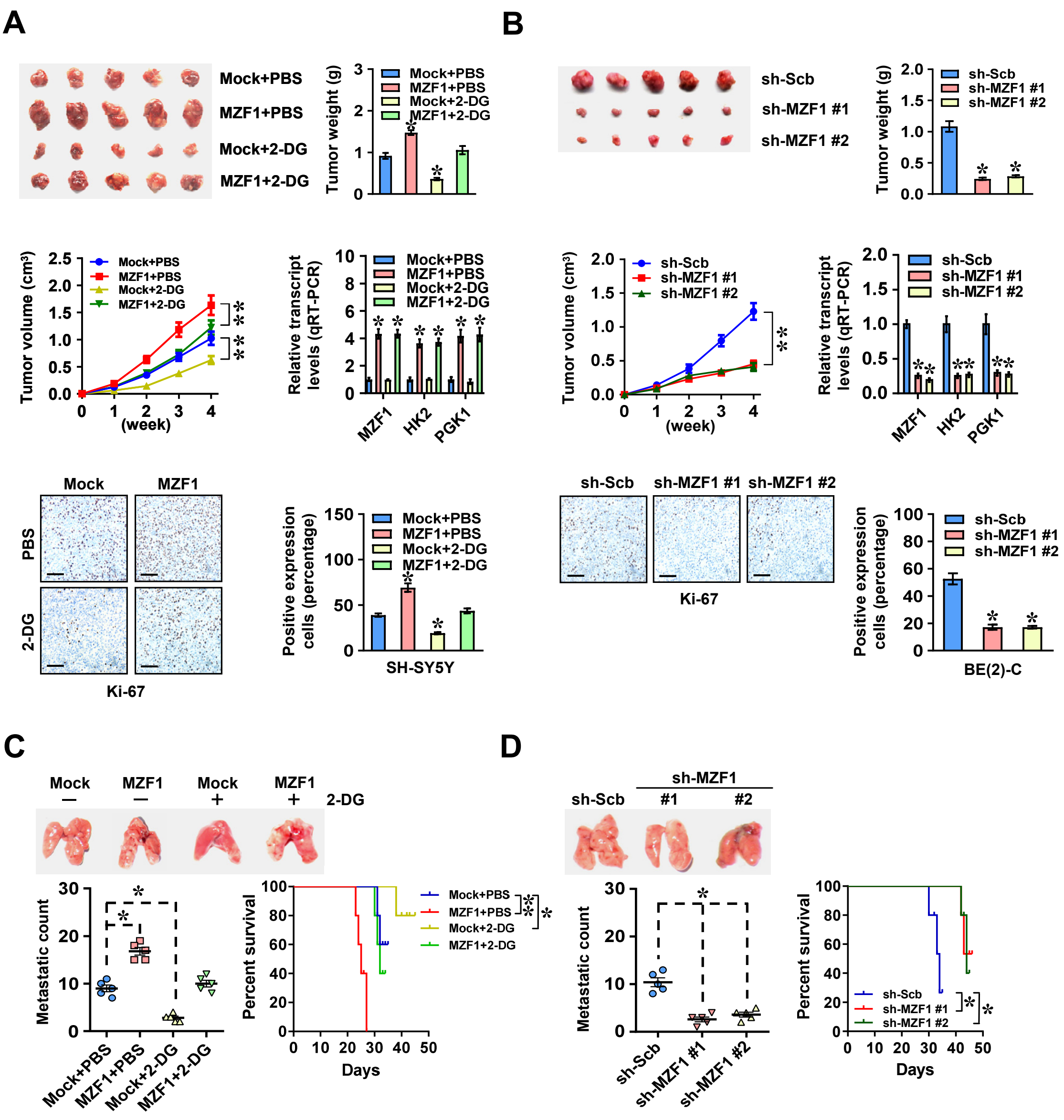

Figure S7. ***MZF1* promotes NB progression via increasing aerobic glycolysis *in vivo*. (A and B)** Representative images, *in vivo* growth curve, weight at the end points, transcript levels of *MZF1*, *HK2* or *PGK1*, images and quantification of Ki-67 immunostaining within subcutaneous xenograft tumors formed by SH-SY5Y (A) or BE(2)-C (B) cells stably transfected with empty vector (mock), *MZF1*, scramble shRNA (sh-Scb), or sh-MZF1 in nude mice, and those treated with daily oral gavage of 2-DG (1 g·kg^{-1}, n=5 for each group). **(C and D)** Representative images (upper panel) and quantification (left lower panel) of lung metastatic colonization and Kaplan-Meier curves (right lower panel) of nude mice treated with tail vein injection of SH-SY5Y (C) or BE(2)-C (D) cells stably transfected with mock, *MZF1*, sh-Scb, or sh-MZF1, and those treated with daily oral gavage of 2-DG (1 g·kg^{-1}, n=5 for each group). ANOVA compared the difference in **A-D**. Log-rank test for survival comparison in **C** and **D**. * P<0.05, ** P<0.01 vs. mock+PBS or sh-Scb. Data are shown as mean ± s.e.m. (error bars) in **A-D**.

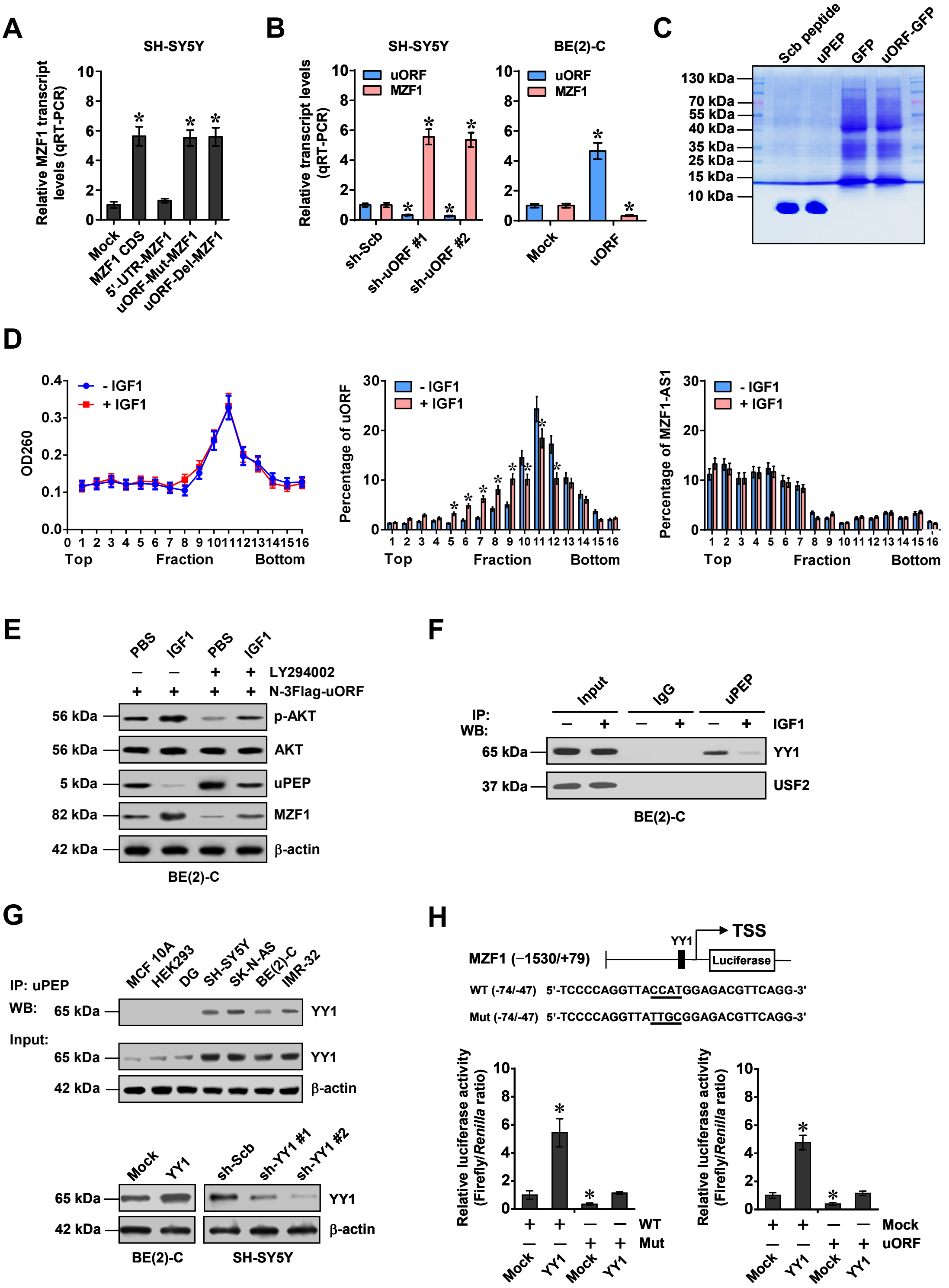

Figure S8. Roles of *MZF1*, *MZF1-uORF*, and *YY1* in NB. (A) Real-time qRT-PCR assay showing the levels of *MZF1* (normalized to β-actin, *n*=4) in SH-SY5Y cells transfected with empty vector (mock), *MZF1* coding sequence (CDS), *MZF1* containing wild-type, mutant, or deletion forms of 5'-UTR, scramble shRNA (sh-Scb), sh-uORF, or *MZF1-uORF*. **(B)** Real-time qRT-PCR assay revealing the levels of *uORF* and *MZF1* (normalized to β-actin, *n*=4) in SH-SY5Y and BE(2)-C cells stably transfected with sh-Scb, sh-uORF, mock, or *uORF*. **(C)** Coomassie blue staining showing the protein expression in BE(2)-C cells transfected with *GFP* or *MZF1-uORF-GFP*, with synthesized scramble (Scb) peptide or uPEP as controls. **(D)** Sucrose gradient sedimentation assay indicating the distribution of *uORF* or *MZF1-AS1* transcript to polysome fractions in SH-SY5Y cells without or with IGF1 (10 $nmol \cdot L^{-1}$) stimulation for 48 hrs. **(E)** Western blot assay showing the levels of p-AKT, uPEP, and MZF1 in BE(2)-C cells stably transfected with 3Flag-tagged *MZF1-uORF* and treated with IGF1 (10 $nmol \cdot L^{-1}$) or LY294002 (10 $\mu mol \cdot L^{-1}$) for 48 hrs. (**F**) Co-IP and western blot assays revealing the interaction of MZF1-uPEP with YY1 or USF2 in BE(2)-C cells with or without IGF1 (10 $nmol \cdot L^{-1}$) treatment for 48 hrs. **(G)** Co-IP and western blot assays indicating the interaction of uPEP with YY1 in non-transformed MCF 10A cells, transformed normal HEK293 cells, normal dorsal root ganglia (DG), and NB cells, and YY1 levels in BE(2)-C and SH-SY5Y cells stably transfected with mock, *YY1*, sh-Scb, or sh-YY. **(H)** Dual-luciferase assay showing the activity of *MZF1* promoter reporter with wild-type (WT) or mutant (Mut) MZF1-binding site in BE(2)-C cells transfected with mock or *YY1*, and those stably transfected with *MZF1-uORF* (*n*=5). ANOVA and Student's *t* test compared the difference in **A**, **B**, **D** and **H**. * $P<0.05$ vs. mock, sh-Scb, -IGF1, or mock+WT. Data are shown as mean ± s.e.m. (error bars) and representative of three independent experiments in **A**-**H**.

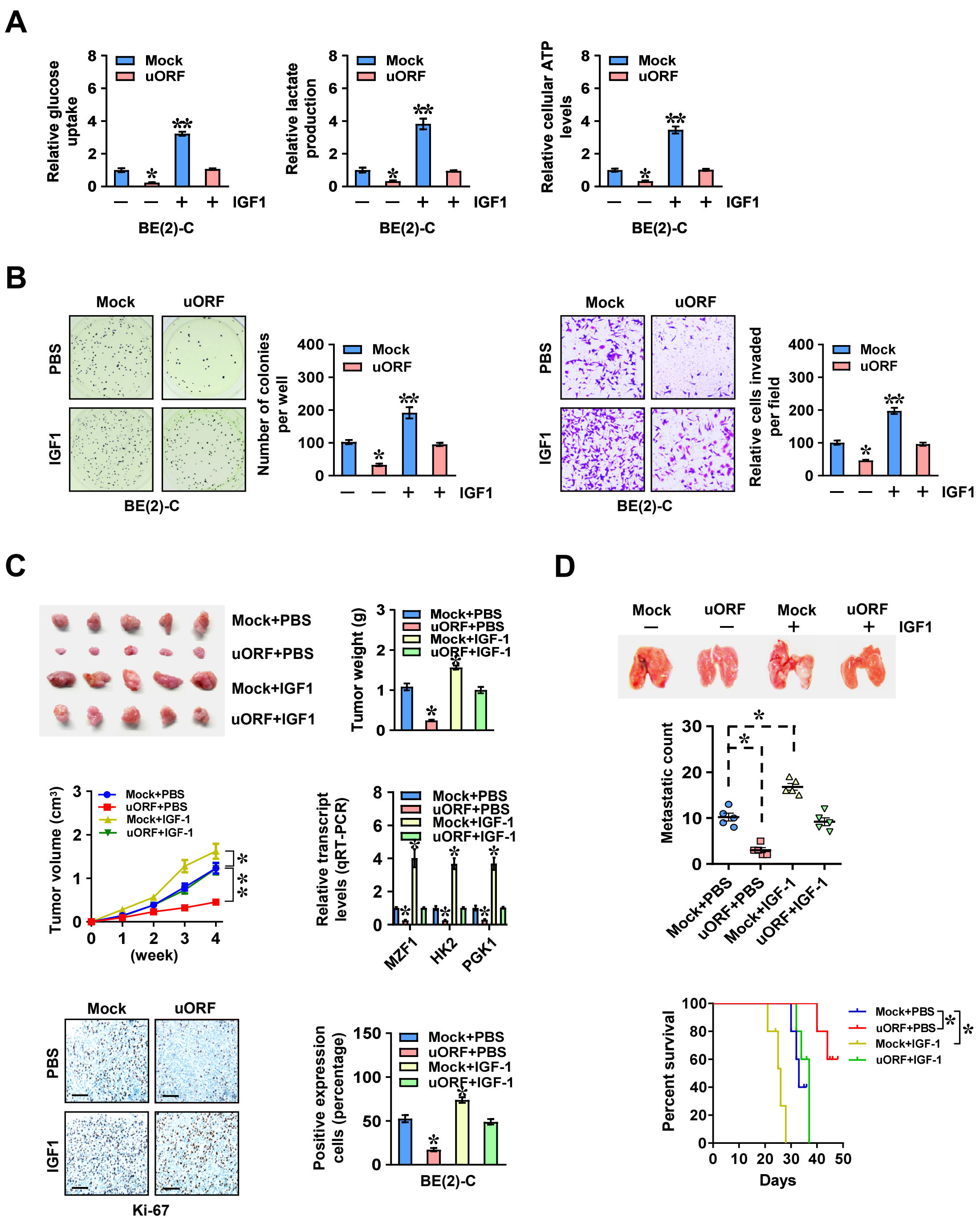

Figure S9. MZF1-uPEP inhibits growth and aggressiveness of NB cells via repressing aerobic glycolysis. (A) Glucose uptake, lactate production, and ATP levels in BE(2)-C (B) cells stably transfected with empty vector (mock) or *MZF1 uORF*, and those treated with IGF1 (10 nmol·L^{-1}) for 48 hrs. **(B)** Representative images (left panel) and quantification (right panel) of soft agar and matrigel invasion assays showing the growth and invasion of BE(2)-C cells stably transfected with mock or *MZF1 uORF*, and those treated with IGF1 (10 nmol·L^{-1}) for 48 hrs. **(C)** Representative images, *in vivo* growth curve, weight at the end points, transcript levels of *MZF1*, *HK2* or *PGK1*, images and quantification of Ki-67 immunostaining within subcutaneous xenograft tumors formed by BE(2)-C cells stably transfected with mock or *MZF1 uORF* in nude mice, and those treated with daily subcutaneous injection of IGF1 (5 mg·kg^{-1}, n=5 for each group). **(D)** Representative images (upper panel) and quantification (middle panel) of lung metastatic colonization and Kaplan-Meier curves (lower panel) of nude mice treated with tail vein injection of BE(2)-C cells stably transfected with mock or *MZF1 uORF*, and those treated with daily subcutaneous injection of IGF1 (5 mg·kg^{-1}, n=5 for each group). ANOVA compared the difference in **A-D**. * P<0.05, ** P<0.01 vs. mock+PBS. Data are shown as mean ± s.e.m. (error bars) in **A-D**.

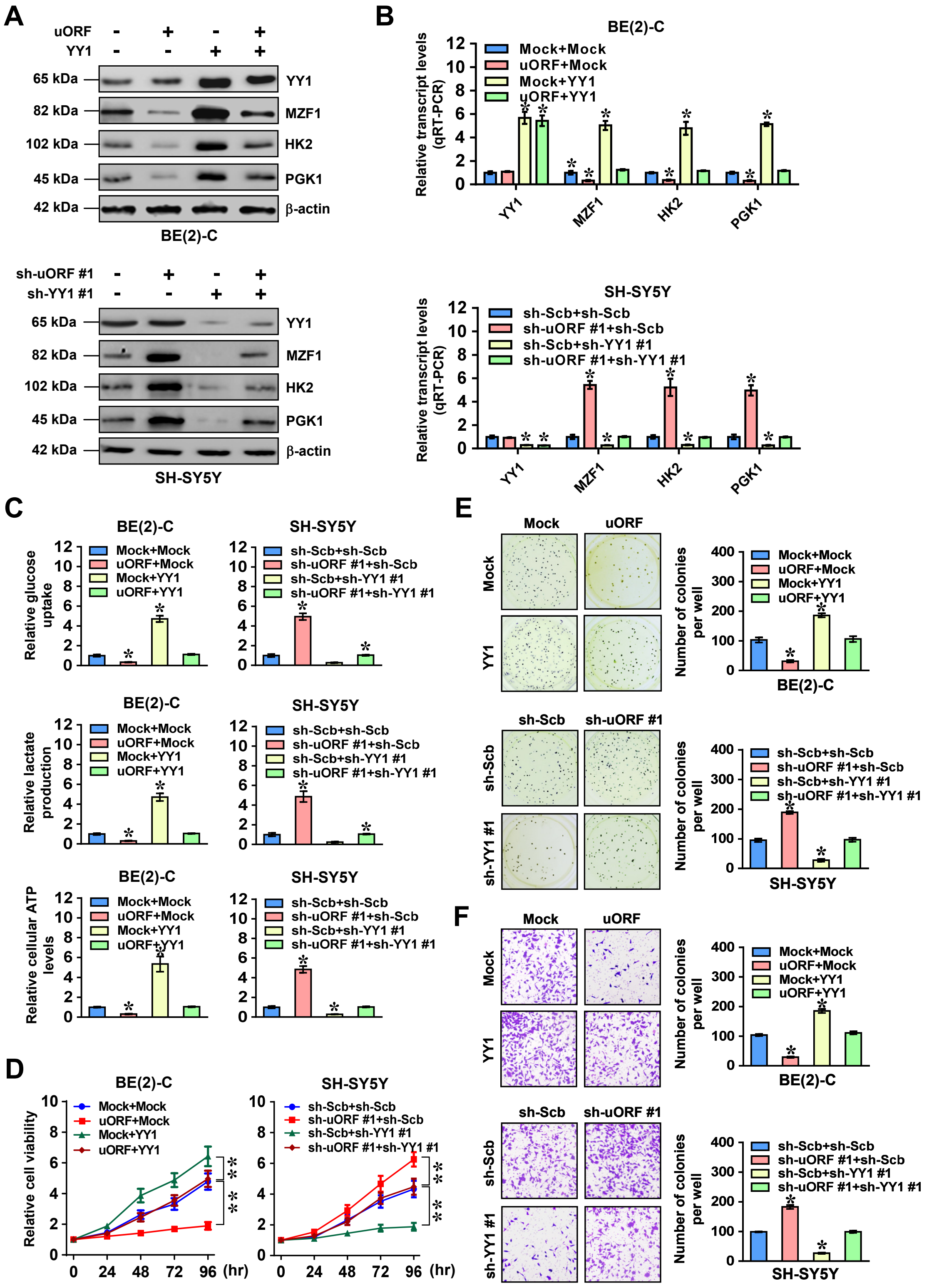

Figure S10. MZF1-uPEP exerts tumor suppressive roles by repressing YY1. (A and B) Western blot (A) and real-time qRT-PCR (B, normalized to β-actin, *n*=4) assays revealing the protein and transcript levels of *YY1*, *MZF1*, *HK2*, and *PGK1* in NB cells stably transfected with empty vector (mock), *MZF1-uORF*, scramble shRNA (sh-Scb), or sh-uORF #1, and those co-transfected with *YY1* or sh-YY1 #1. **(C)** Glucose uptake, lactate production, and ATP levels in NB cells stably transfected with mock, *MZF1-uORF*, sh-Scb, or sh-uORF #1, and those co-transfected with *YY1* or sh-YY1 #1 (*n*=4). **(D)** MTT colorimetric assay showing the viability of BE(2)-C and SH-SY5Y cells stably transfected with mock, *MZF1-uORF*, sh-Scb, or sh-uORF #1, and those co-transfected with *YY1* or sh-YY1 #1 (*n*=6). **(E and F)** Representative images (left panel) and quantification (right panel) of soft agar (E) and matrigel invasion (F) assays indicating the anchorage-independent growth and invasion capability of NB cells stably transfected with mock, *MZF1-uORF*, sh-Scb, or sh-uORF #1, and those co-transfected with *YY1* or sh-YY1 #1 (*n*=4). ANOVA compared the difference in **B-F**. * *P*<0.05, ** *P*<0.01 vs. mock or sh-Scb. Data are shown as mean ± s.e.m. (error bars) and representative of three independent experiments in **A-F**.

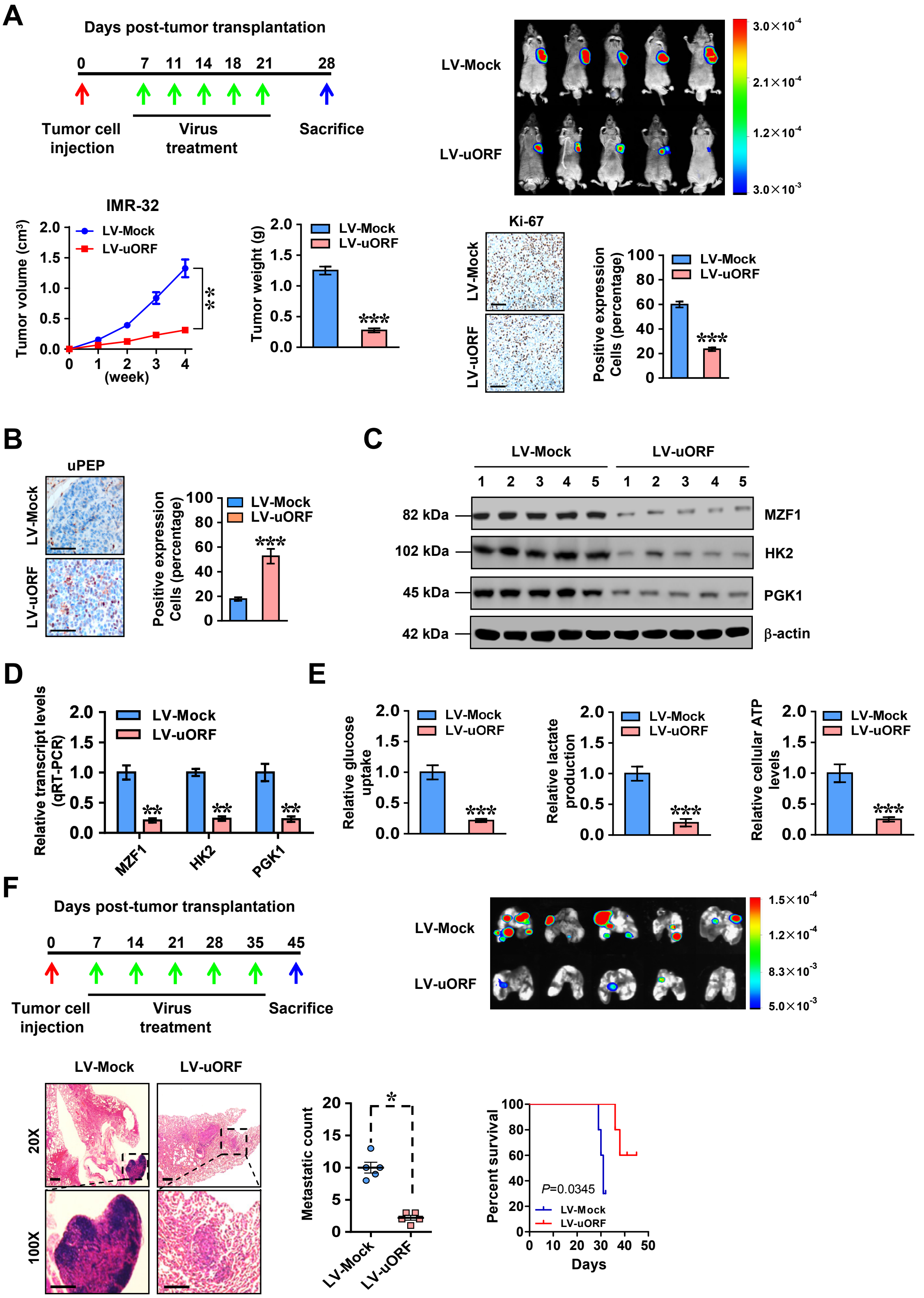

Figure S11. Therapeutic over-expression of *MZF1-uORF* inhibits NB progression. (A) *In vivo* imaging (upper right panel), growth curve (lower left panel), representative images and weight at the end points (lower middle panel), and immunohistochemical staining of Ki-67 (lower right panel) of IMR-32 cells-formed subcutaneous xenograft tumors in athymic nude mice (n=5 per group) that received tail vein injection of lentivirus (LV)-mediated empty vector (mock) or *MZF1-uORF* as indicated (upper left panel). Scale bar: 100 μm. **(B-D)** Immunohistochemical staining (B), western blot (C) and real-time qRT-PCR (D, normalized to β-actin) assays indicating the expression of MZF1-uPEP and downstream genes within IMR-32 cells-formed subcutaneous xenograft tumors in nude mice that received tail vein injection of LV-mediated mock or *MZF1-uORF* (n=5 per group). Scale bar: 100 μm. **(E)** Glucose uptake, lactate production, and ATP levels of IMR-32 cells-formed subcutaneous xenograft tumors in nude mice (n=5 per group) that received intravenous injection of LV-mediated mock or *MZF1-uORF*. **(F)** LV-mediated therapy timeline (upper left panel), *in vivo* imaging of lungs (upper right panel), representative images and metastatic counts of lungs (lower left and middle panels), and Kaplan-Meier curves (lower right panel) of nude mice (n=5 per group) treated with tail vein injection of IMR-32 cells and LV-mediated mock or *MZF1-uORF*. Scale bar: 100 μm. Student's t test and ANOVA compared the difference in **A**, **B** and **D-F**. Log-rank test for survival comparison in **F**. * P<0.05, ** P<0.01, *** P<0.001 vs. LV-Mock. Data are shown as mean ± s.e.m. (error bars).

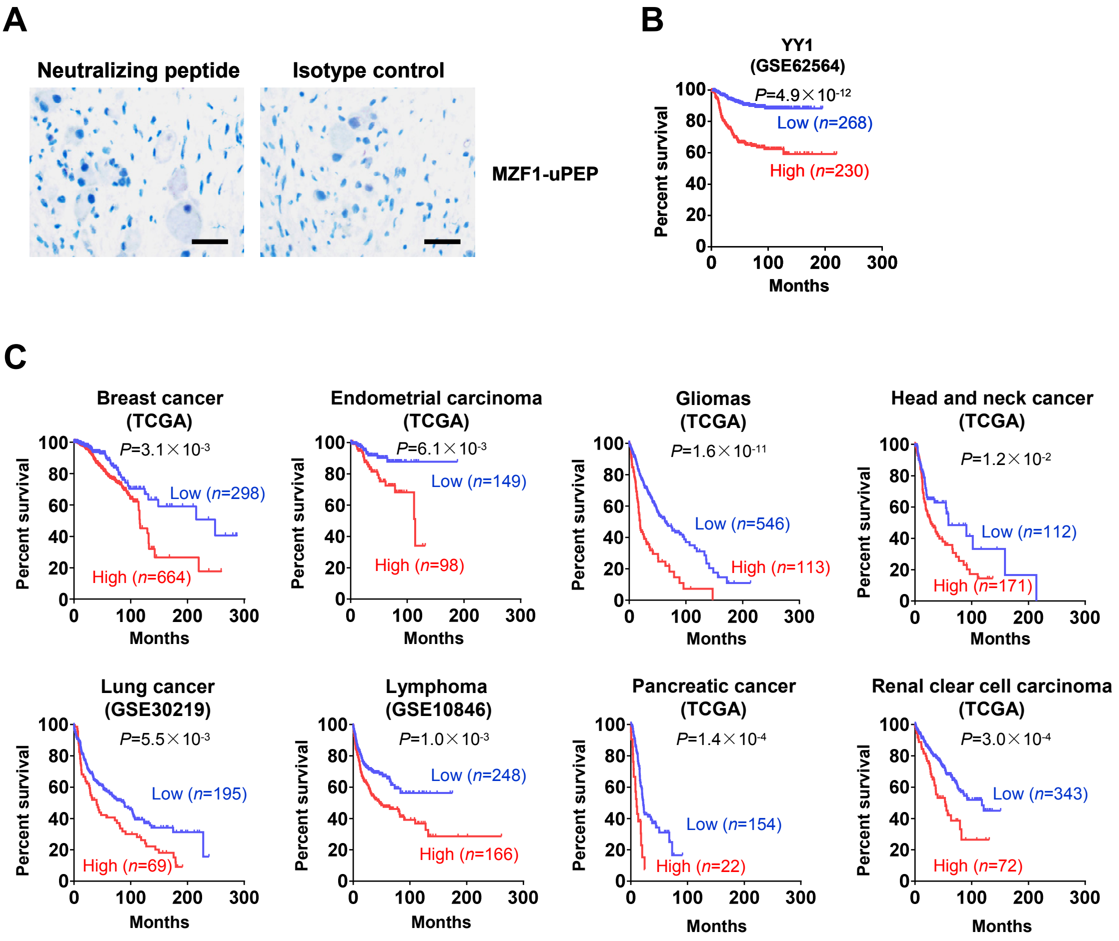

Figure S12. Expression of *MZF1-uPEP* and *YY1* in NB tissues. (A) Representative images of immunohistochemical staining showing the expression of MZF1-uPEP in NB specimens incubated with neutralizing antigen peptide or IgG isotype control. Scale bars: 50 μm. **(B)** Kaplan-Meier curve showing the overall survival of 498 NB patients (GSE62564) with high or low *YY1* expression (cutoff value=47.2). **(C)** Kaplan–Meier survival plots of patients with low or high levels of *YY1* in TCGA or GEO datasets of breast cancer, endometrial carcinoma, glioma, head and neck carcinoma, lung cancer, lymphoma, pancreatic cancer, or renal clear cell carcinoma. Log-rank test for survival comparison in **B** and **C**.

Table S1 Primer sets used for qRT-PCR and ChIP

Primer set	Primers	Sequence	Product size	Application
MZF1	Forward	5'-CTGAAACTGAGCCTCCAACTCC-3'	232	qRT-PCR
	Reverse	5'-CCAGTCTTGCTGTGGGGAAA-3'		
MZF1-uORF	Forward	5'-ATGGAGACGCGGTGGGGCA-3'	63	qRT-PCR
	Reverse	5'-GCAGGATCCTGCACCAATCA-3'		
ALDOC	Forward	5'-ATGGTGTTCCCTTCGTCCGA-3'	114	qRT-PCR
	Reverse	5'-GCCCTTGAGTGGTGGTTTCT-3'		
ENO1	Forward	5'-GATGACTGGGGAGCTTGGCAGAAG-3'	134	qRT-PCR
	Reverse	5'-TTGAGCAGGAGGCAGTTGCAGGAC-3'		
GPI	Forward	5'-AGTTCTGGGATTGGGTGGGA-3'	230	qRT-PCR
	Reverse	5'-ATAGGGCAGCATGGCGTGTG-3'		
HK2	Forward	5'-TGGAGCGAGGTCTGAGCAAG-3'	145	qRT-PCR
	Reverse	5'-ACCAGCAGGACCCGGAAATT-3'		
LDHA	Forward	5’-TGTCTCTGGCAAAGTGGAT-3	117	qRT-PCR
	Reverse	5’-ATTAGGTAACGGAATCGGG-3		
PGK1	Forward	5'-AGCCAAGATTGTCAAAGACCT-3'	255	qRT-PCR
	Reverse	5'-GCTTCCCATTCAAATACCCC-3'		
MZF1-AS1	Forward	5'-GGAATCTGCGTTTATGAAGGGGA-3'	197	qRT-PCR
	Reverse	5'-TTCTGGGAAGTGTATTTCGGGAC-3'		
β-actin	Forward	5'-TGCCCATCTACGAGGGGTATG-3'	156	qRT-PCR
	Reverse	5'-TCTCCTTAATGTCACGCACGATTT-3'		
HK2 (-467/-290)	Forward	5’-AATACCCCAGCATTTACCAATA-3’	178	ChIP
	Reverse	5’-CTGCCCTTGTCTTACTCCTTCT-3’		
PGK1 (-1507/-1346)	Forward	5’-ATTGATAACAGCCCTTTCCCCTTCT-3’	162	ChIP
	Reverse	5'-ATGGTGATGTTATTCCCAGAAGCAA-3'		
MZF1 (-156/+32)	Forward	5’-CCCCACCTCTTCATCCCACAT-3’	188	ChIP
	Reverse	5’-GTAGTCAGCAGGCCCTTCGTG-3’		

MZF1, myeloid zinc finger 1; uORF, upstream open reading frame; ALDOC, aldolase, fructose-bisphosphate C; ENO1, enolase 1; GPI, glucose-6-phosphate isomerase; HK2, hexokinase 2; LDHA, lactate dehydrogenase A; PGK1, phosphoglycerate kinase 1; MZF1-AS1, myeloid zinc finger 1 antisense RNA 1.

Table S2 Oligonucleotide sets used for constructs

Primer set	Primers	Sequence
dCas9a-MZF1 #1	Forward	5'-CACCGGACACGACTCGACATCGCCTGGG-3'
	Reverse	5'-AAACCCCAGGCGATGTCGAGTCGTGTCC-3'
dCas9a-MZF1 #2	Forward	5'-CACCGGGGTAACCCGGAGACGCGGCGGG-3'
	Reverse	5'-AAACCCCGCCGCGTCTCCGGGTTACCCC-3'
dCas9i-MZF1 #1	Forward	5'-CACCGGGCCGTCATTAGAACTCCGTCGG-3'
	Reverse	5'-AAACCCGACGGAGTTCTAATGACGGCCC-3'
dCas9i-MZF1 #2	Forward	5'-CACCGCACCGACGGAGTTCTAATGACGG-3'
	Reverse	5'-AAACCCGTCATTAGAACTCCGTCGGTGC-3'
pGL3-5'-UTR	Forward	5'-GGAAGATCTGCGCACGTACACACGAAGGGCCTG-3'
	Reverse	5'-CCCAAGCTTAGAGGGTACCAGGTCAGGTATCTG-3'
pGL3-P-5'-UTR	Forward	5'-GGAAGATCTTCACAGTGCAGTTTTCCGGAGGACG-3'
	Reverse	5'-CCCAAGCTTAGAGGGTACCAGGTCAGGTATCTG-3'
pGL3-5'-UTR Mut	Forward	5'-GGGAGTTGCCTAGGAGACGCGGTGGGCACCGACGGAGTTCTA-3'
	Reverse	5'-ACCGCGTCTCCTAGGCAACTCCCTACAGGAAGGGGCCTCTGCT-3'
pGL3-5' UTR Del	Forward	5'-AGTTGCCATGAGACGCGGTGGGGCACCGACGGAGTTCTAAT-3'
	Reverse	5'-CCACCGCGTCTCATGGCAACTCCCTACAGGAAGGGGCCTCT-3'
pcDNA3.1-MZF1 CDS	Forward	5'-CCCAAGCTTATGAGGCCTGCGGTGCTGGGCT-3'
	Reverse	5'-CCGGAATTCCTACTCGGCGCTGTGGACGCGC-3'
pcDNA3.1-5'-UTR-MZF1	Forward	5'-CCCAAGCTTGCGCACGTACACACGAAGGGCC-3'
	Reverse	5'-CCGGAATTCGGGTGGAGACCCAGTTTCCATC-3'
pcDNA3.1-5'-UTR-MZF1 Mut	Forward	5'-GGGAGTTGCCTAGGAGACGCGGTGGGGCACCGACGGAGTTCTA-3'
	Reverse	5'-ACCGCGTCTCCTAGGCAACTCCCTACAGGAAGGGGCCTCTGCT-3'
pcDNA3.1-5'-UTR-MZF1 Del	Forward	5'-AGTTGCCATGAGACGCGGTGGGGCACCGACGGAGTTCTAAT-3'
	Reverse	5'-CCACCGCGTCTCATGGCAACTCCCTACAGGAAGGGGCCTCT-3'
pCMV-3Tag-1A-MZF1 uORF	Forward	5'-CCCAAGCTTATGGAGACGCGGTGGGGCA-3'
	Reverse	5'-GCCGCTCGAGTTAGCAGGATCCTGCACCAA-3'
pCMV-C-Tag-MZF1 uORF	Forward	5'-CCCAAGCTTATGGAGACGCGGTGGGGCA-3
	Reverse	5'-GCCGCTCGAGGCAGGATCCTGCACCAATCA-3
pEGFP-N1-MZF1-uORF	Forward	5'-GCCGCTCGAGATGGAGACGCGGTGGGGCA-3
	Reverse	5'-CCCAAGCTTGCAGGATCCTGCACCAATCA-3'
pEGFP-N1-MZF1 uORF Mut	Forward	5'-AGATCTCGAGTAGGAGACGCGGTGGGGCACCGACGGAGTTCTA-3'
	Reverse	5'-ACCGCGTCTCCTACTCGAGATCTGAGTCCGGTAGCGCTAGCGG-3'
pEGFP-N1-MZF1 uORF Del	Forward	5'-TCTCGAGATGAGACGCGGTGGGGCACCGACGGAGTTCTAAT-3'
	Reverse	5'-CCACCGCGTCTCATCTCGAGATCTGAGTCCGGTAGCGCTAG-3'
pEGFP-N1 Mut	Forward	5'-ACCGGTCGCCTCCCTGTTGAGCAAGGGCGAGGAGCTGTTCACCGGGGT-3'
	Reverse	5'-GCCCTTGCTCAACAGGGAGGCGACCGGTGGATCCCGGGCCCGCGGTAC-3'
pCMV-N-MYC-YY1	Forward	5'-CCGGAATTCATGGCCTCGGGCGACACCCTCTA-3'
	Reverse	5'-GCCGCTCGAGTCACTGGTTGTTTTTGGCCTTAG-3'
pCMV-N-MYC-YY1 (N-terminal)	Forward	5'-CCGGAATTCATGGCCTCGGGCGACACCCTCTA-3'
	Reverse	5'-GCCGCTCGAGCGGGTCGGCGCCGCCGCCGCCCG-3'
pCMV-N-MYC-YY1 (ΔZFN)	Forward	5'-CCGGAATTCATGGCCTCGGGCGACACCCTCTA-3'
	Reverse	5'-GCCGCTCGAGTGTCATATATTCTGAATAATCAG-3'
pCMV-N-MYC-YY1 (Δ-N-terminal)	Forward	5'-CCGGAATTCGGCAACAAGAAGTGGGAGCAGAA-3'
	Reverse	5'-GCCGCTCGAGTCACTGGTTGTTTTTGGCCTTAG-3'
pCMV-N-MYC-YY1 (C-terminal)	Forward	5'-CCGGAATTCGAAAAAAAAGATATTGACCATGA-3'
	Reverse	5'-GCCGCTCGAGTCACTGGTTGTTTTTGGCCTTAG-3'
pLV-MZF1 uORF	Forward	5'-TGCTCTAGAATGGATTACAAGGATGACGACG-3'
	Reverse	5'-ATTTGCGGCCGCTTAGCAGGATCCTGCACCAATC-3'
pGL3-YY1	Forward	5'-CCGGCCATCTTGATACGACCATCTTCTATCGGCCATCTTGATACGACCATGTTCA-3'
	Reverse	5'-AGCTTGAACATGGTCGTATCAAGATGGCCGATAGAAGATGGTCGTATCAAGATGGCCGGGTAC-3'
pGL3-MZF1 (-1530/+30)	Forward	5'-CGGGGTACCTCCCGGGTTCACGCCATTCTCCTGC-3'
	Reverse	5'-GCCGCTCGAGTAGACCGCAAAATGGAGAAAGGCTT-3'
pGL3-MZF1 Mut	Forward	5'-CCCCAGGTTATTGCGGAGACGTTCAGGGCCCGCCCCTCGCCCATG-3'
	Reverse	5'-TGAACGTCTCCGCAATAACCTGGGGACACGACTCGACATCGCCTG-3'
pGL3-HK2 (-1813/+424)	Forward	5'-CAAGGAAAGCCTGATGAGGTAGAAG-3'
	Reverse	5'-TGATGATGTGAATGAACTGGGTAGA-3'
pGL3-HK2 Mut	Forward	5'-TCAGGAGCCTAGATAGTGAGGTTAGCCAGAAACCCTCAGAGTGGA-3'
	Reverse	5'-CTAACCTCACTATCTAGGCTCCTGAGTCCCTGTTTGCTCTATTGG-3'
pGL3-PGK1 (-882/+246)	Forward	5'-CGGGGTACCCTAAGAACTTGGACACCCTCCACG-3'
	Reverse	5'-GCCGCTCGAGCAGAATTACCTCATAACGACCCGC-3'
pGL3-PGK1 (Mut)	Forward	5'-CGACCTCTCTACTGAGCTGTATTTCCAAAATGTCGCTTTCTAACA-3'
	Reverse	5'-GAAATACAGCTCAGTAGAGAGGTCGGTGATTCGGTCAACGAGGGA-3'

MZF1, myeloid zinc finger 1; uORF, upstream open reading frame; YY1, Yin Yang 1; HK2, hexokinase 2; PGK1, phosphoglycerate kinase 1.

Table S3 Oligonucleotides encoding short hairpin RNAs

Oligo Set	Sequences
sh-Scb	5'-CCGGGCGAACGATCGAGTAAACGGACTCGAGTCCGTTTACTCGATCGTTCGCTTTTT-3' (sense);
	5'-AATTCAAAAAGCGAACGATCGAGTAAACGGACTCGAGTCCGTTTACTCGATCGTTCGC-3' (antisense)
sh-MZF1 #1	5'-CCGGCCGGCCTGCCTTTGACCCTATCTCGAGATAGGGTCAAAGGCAGGCCGGTTTTTG-3' (sense);
	5'-GATCCAAAAACCGGCCTGCCTTTGACCCTATCTCGAGATAGGGTCAAAGGCAGGCCGG-3' (antisense)
sh-MZF1 #2	5'-CCGGGACTGGGAGAGAATGCCCAGTCTCGAGACTGGGCATTCTCTCCCAGTCTTTTTG-3' (sense);
	5'-GATCCAAAAAGACTGGGAGAGAATGCCCAGTCTCGAGACTGGGCATTCTCTCCCAGTC-3' (antisense)
sh-HK2 #1	5'-CCGGTGGAGCTCAACCATGACCAAGTCTCGAGACTTGGTCATGGTTGAGCTCCTTTTTG-3' (sense);
	5'-GATCCAAAAAGGAGCTCAACCATGACCAAGTCTCGAGACTTGGTCATGGTTGAGCTCCA-3' (antisense)
sh-HK2 #2	5'-CCGGTGGGTGAAAGTAACGGACAATGCTCGAGCATTGTCCGTTACTTTCACCCTTTTTG-3' (sense);
	5'-GATCCAAAAAGGGTGAAAGTAACGGACAATGCTCGAGCATTGTCCGTTACTTTCACCCA-3' (antisense)
sh-PGK1 #1	5'-CCGGTGCTGTCCCAAGCATCAAATTCCTCGAGGAATTTGATGCTTGGGACAGCTTTTTG-3' (sense);
	5'-GATCCAAAAAGCTGTCCCAAGCATCAAATTCCTCGAGGAATTTGATGCTTGGGACAGCA-3' (antisense)
sh-PGK1 #2	5'-CCGGTGCCTGACAAGTACTCCTTAGACTCGAGTCTAAGGAGTACTTGTCAGGCTTTTTG-3' (sense);
	5'-GATCCAAAAAGCCTGACAAGTACTCCTTAGACTCGAGTCTAAGGAGTACTTGTCAGGCA-3' (antisense)
sh-MZF1-uORF #1	5'-CCGGGCAGCTTCATAACCGAAGATTCTCGAGAATCTTCGGTTATGAAGCTGCTTTTTG-3' (sense);
	5'-GATCCAAAAAGCAGCTTCATAACCGAAGATTCTCGAGAATCTTCGGTTATGAAGCTGC-3' (antisense)
sh-MZF1-uORF #2	5'-CCGGCCGAGAAATCTCTTACCTCAACTCGAGTTGAGGTAAGAGATTTCTCGGTTTTTG-3' (sense);
	5'-GATCCAAAAACCGAGAAATCTCTTACCTCAACTCGAGTTGAGGTAAGAGATTTCTCGG-3' (antisense)
sh-YY1 #1	5'-CCGGGCTTCACATATCAGCAGGTTACTCGAGTAACCTGCTGATATGTGAAGCTTTTTG-3' (sense);
	5'-GATCCAAAAAGCTTCACATATCAGCAGGTTACTCGAGTAACCTGCTGATATGTGAAGC-3' (antisense)
sh-YY1 #2	5'-CCGGACCTCTTCGACTGTGACTTTGCTCGAGCAAAGTCACAGTCGAAGAGGTTTTTTG-3' (sense);
	5'-GATCCAAAAAACCTCTTCGACTGTGACTTTGCTCGAGCAAAGTCACAGTCGAAGAGGT-3' (antisense)

MZF1, myeloid zinc finger 1; HK2, hexokinase 2; PGK1, phosphoglycerate kinase 1; uORF, upstream open reading frame; YY1, YY1 transcription factor.

Table S4 Glycolytic genes and transcription factors differentially expressed in NB (GSE16476)

Differentially expressed glycolytic genes

Age	Death	2 vs. 4 stage	Overlap
ALDOA	ALDOA	ALDOC	ALDOC
ALDOC	ALDOC	ENO1	ENO1
ENO1	ENO1	GPI	GPI
GPI	GPI	HK2	HK2
HK2	HK2	LDHA	LDHA
LDHA	LDHA	PGK1	PGK1
PFKM	PGK1	PKM	PKM
PGK1	PKM		
PKM	SLC2A1		

Differentially expressed transcription factors

Age	Death		2 vs. 4 stage	Overlap
ATF2	AATF	ZNF138	CBFB	CBFB
BUD31	AFF4	ZNF219	E2F3	E2F3
CBFA2T2	ATF2	ZNF256	E2F8	HMGA1
CBFB	CBFA2T2	ZNF287	GATAD2A	MZF1
CREB1	CBFB	ZNF367	HMGA1	TRIM28
E2F3	CREB1	ZNF496	MYCN	ZEB2
ENO1	CREB5	ZNF70	MZF1	ZHX2
FOXP1	CREBL2	ZNF83	NR3C1	ZNF496
HMGA1	DLX5	ZNF93	PLAGL2	ZNF93
HOXC9	DMRT3	ZRANB2	SCML2	
KLF5	E2F1		TRIM28	
KLF7	E2F3		UHRF1	
MZF1	E2F6		ZEB2	
NFYA	E2F8		ZHX2	
NR3C1	ENO1		ZNF496	
PA2G4	ESRRA		ZNF93	
RORA	ETV3			
SALL1	ETV4			
SCAND1	FEV			
SOX6	FOXC1			
TCF7L2	FOXD3			
TRIM28	FOXM1			
TSHZ2	FOXO3			
TULP4	FOXP1			
VAX2	FUBP1			
VPS72	GATAD2A			
ZBTB38	HES6			
ZEB2	HIF1A			
ZFP36L2	HMGA1			
ZHX2	HMGB2			
ZNF138	HMX1			
ZNF219	HOXC9			
ZNF256	HOXD10			
ZNF496	HOXD4			
ZNF93	HOXD9			
	IRX3			
	IRX5			
	JUN			
	JUND			
	KLF12			
	KLF5			
	KLF7			
	MTA1			
	MYBL2			
	MYT1L			
	MZF1			
	NFATC3			
	NFYA			
	NR2C1			
	NR2F1			
	PA2G4			
	PLAGL2			
	POU4F1			
	POU6F1			
	PTTG1			
	RELA			
	RFXANK			
	RNF4			
	RORA			
	RORB			
	SCML2			
	SIX2			
	SMAD3			
	SMARCA4			
	SNAPC2			
	SOX4			
	SOX6			
	SRF			
	TBX1			
	TCF3			
	TEAD4			
	THRA			
	TLX3			
	TP53			
	TRIM28			
	TSHZ2			
	TULP4			
	UHRF1			
	VAX2			
	YY1			
	ZBTB38			
	ZEB2			
	ZHX2			

Table S5 Mass spectrometry analysis of proteins pulled down by MZF1-uPEP

A1BG	CACYBP	DHDDS	GNPDA2	LPL	NUP160	PTP4A1	SMAD1	TUBB2B
ABCA1	CADM4	DHFR	GNPNAT1	LRRC40	NUP188	PTP4A2	SMAD2	TUBG1
ABCC5	CALCOCO2	DHODH	GNS	LRRC47	NUP43	PTPN1	SMAD3	TUBG2
ABCF3	CAMK1	DHPS	GOLGB1	LRRC57	NUP50	PTPRA	SMAD4	TUBGCP4
ABHD18	CAMK2A	DHRS1	GOLIM4	LRRN2	NUP54	PTPRK	SMAD5	TUFM
ACACA	CAPN1	DHX29	GOLPH3	LSM14B	NUP62	PTRF	SMAD9	TWF2
ACAD9	CAPZA1	DHX36	GPAT2	LSM2	NUP85	PUM1	SMARCAD1	TXNDC12
ACADS	CARF	DHX38	GPD1	LSM3	NUTM2F	PUS10	SMARCE1	U2AF1
ACBD3	CARM1	DHX57	GPR135	LSM4	NUTM2G	PUS7	SMC3	U2AF1L5
ACOT9	CARNMT1	DIAPH3	GRHPR	LTA4H	NXF2	PWP2	SMC5	U2AF2
ACP1	CASP8AP2	DIMT1	GRIN3B	LUC7L3	NXF2B	PYGB	SMPD4	UAP1
ACSL1	CAT	DIP2A	GRIPAP1	LUZP1	ODR4	QDPR	SMS	UBA6
ACTA1	CBX5	DIS3	GRPEL1	LYAR	OGT	QKI	SMYD5	UBE2C
ACTA2	CC2D2A	DISC1	GRWD1	LZTR1	OR1B1	RAB11FIP2	SNAP23	UBE2D1
ACTC1	CCAR1	DKFZp566H1924	GSTM1	LZTS3	OR2I1P	RAB12	SNAPIN	UBE2D4
ACTG2	CCAR2	DKFZp686E0752	GSTM3	MAATS1	OSBP	RAB14	SNCG	UBE2E1
ACTN3	CCBL2	DKFZp686I0230	GSTM4	MAN2A1	OSBPL7	RAB21	SNF8	UBE2E2
ACTR10	CCDC141	DLD	GTF2I	MAP1LC3B	OSBPL8	RAB23	SNRPC	UBE2E3
ACTR5	CCDC157	DLGAP4	GTPBP1	MAP1LC3B2	OSBPL9	RAB3A	SNRPG	UBE2G2
ADAM28	CCDC171	DMXL1	GTSE1	MAP2	OSTF1	RAB3B	SNRPGP15	UBE2I
ADARB1	CCDC173	DNAH3	GUK1	MAP3K14	OTUD6B	RAB3C	SNTB2	UBE2K
ADH6	CCDC47	DNAH6	GYS2	MAP4K4	OXSR1	RAB3D	SNX1	UBE2L3
ADH7	CCDC79	DNAH9	HADH	MAP7D3	P2RX4	RAB3GAP2	SNX25	UBE2O
ADRA1D	CCDC85C	DNAJB1	HAT1	MAP9	P3H1	RAB41	SNX6	UBE2T
ADRA2B	CCDC87	DNAJB11	HCCS	MARC2	PACSIN2	RAB44	SNX8	UBE3A
ADRM1	CCNE1	DNAJC10	HDAC2	MARS	PAFAH1B2	RAB5B	SOS2	UBE3C
ADSL	CDC123	DNAJC19	HEATR1	MAT2B	PAIP1	RAB5C	SPAG16	UBE4A
AGK	CDC73	DNAJC21	HECTD1	MCF2L	PARP10	RAB6B	SPATA31D1	UBE4B
AGL	CDCA7L	DNAJC7	HECTD4	MCTS1	PARVA	RAB8A	SPATA31D3	UBR3
AGPAT5	CDIPT	DNM1L	HEL-S-109	MDN1	PBDC1	RAB8B	SPATA31D4	UBR5
AGPS	CDK2	DNMT1	HELZ	ME1	PCBD1	RAD1	SPCS2	UBTF
AHR	CDK5	DPH7	HGS	MEAF6	PCBD2	RAD21	SPIRE1	UBXN2B
AIF1L	CDK5R2	DPM1	HGSNAT	MEPE	PCBP3	RAE1	SPIRE2	UBXN4
AIMP2	CDKN2AIPNL	DPP10	HID1	MESDC2	PCDHAC2	RAI1	SPNS1	UBXN7
AKAP2	CEBPE	DPP9	HIRA	METTL22	PCDHB11	RALGAPA2	SPOPL	UFC1
AKAP8L	CEBPZ	DR1	HLA-B	MGAT2	PCDHB14	RARS	SQSTM1	UFL1
ALDH3A2	CELF2	DRG2	HLA-C	MIC13	PCDHGB3	RASA3	SRC	UFM1
ALDH3B1	CEP135	DSTN	HM13	MIDN	PCYT2	RASAL2	SRP14	UGP2
ALDH3B2	CEP162	DTNBP1	HMGB2	MIEN1	PDCD1	RASIP1	SRP54	UGT2B15
ALDH9A1	CEP95	DUPD1	HMGB3	MLEC	PDCD11	RASSF5	SRPRA	UHMK1
ALF	CERS2	DUS3L	HMGCL	MOB1A	PDCL3	RASSF7	SRPRB	UHRF1
ALYREF	CFAP36	DUSP15	HMGCS1	MOB1B	PDE4DIP	RAVER1	SRSF10	UHRF1BP1
AMPH	CFAP47	DYNC1I2	HMOX2	MOB4	PDK3	RBBP7	SRSF11	UHRF1BP1L
ANAPC7	CFAP54	DYNC1LI1	HNRNPA0	MOSPD2	PDLIM2	RBM12	SSB	UHRF2
ANGPT4	CFAP58	DYNLT1	HNRNPD	MPRIP	PDLIM5	RBM22	SSH3	UMODL1
ANKHD1	CHAF1B	ECM29	HNRNPH3	MRC2	PDP2	RBM24	SSR1	UNC119
ANKRD17	CHD2	EEF1E1	HNRNPUL1	MRE11A	PDS5A	RBM25	SSR3	UNC119B
ANKRD66	CHD3	EEFSEC	HPCA	MROH2A	PDS5B	RBM28	SSR4	UNC13C
ANKRD7	CHD5	EFCAB8	HPCAL1	MROH5	PDZD2	RBM38	ST13P5	UNC13D
ANKUB1	CHD7	EHBP1	HSFY1	MRPL17	PEAK1	RBM4	ST6GAL2	UNC79
ANO4	CHKB	EHD4	HSP90AB3P	MRPL19	PELP1	RBPJ	STK10	UNKL
ANO6	CHMP6	EIF2B2	HSP90B2P	MRPL24	PEPD	RBX1	STK17A	UQCRB
ANP32A	CHRNB1	EIF3G	HSPA14	MRPL32	PEX1	RCC1	STOM	USF2
ANP32D	CHURC1-FNTB	EIF3K	HSPA1A	MRPL9	PEX14	RDH11	STON1-GTF2A1L	USP10
ANXA11	CIAPIN1	EIF3M	HSPA1B	MRPS16	PFAS	RDH13	STRN	USP14
ANXA13	CISD1	EIF4A2	HSPE1-MOB4	MRPS18B	PFDN5	RDH14	STT3B	USP32
ANXA7	CISD2	EIF4G3	IARS2	MRPS22	PFKM	REEP4	STX4	USP39
AP1M1	CKS1B	EIF4H	IDI1	MRPS23	PGD	REEP5	STXBP1	USP4
AP2S1	CLASP2	EIF6	IER3IP1	MRPS34	PGK2	RFC5	STXBP4	USP9Y
AP3D1	CLDND1	ELAC2	IFIT1	MRTO4	PGLS	RHOC	STXBP6	UTP4
AP3M1	CLEC16A	ELMO2	IFITM1	MSANTD4	PGM2	RIC1	SUPT5H	UTRN
AP3S1	CLIC2	EMC2	IFITM2	MSTO1	PGS1	RILPL1	SUZ12	UVRAG
AP4B1	CLIP1	ENTPD3	IFITM3	MT-CYB	PHF2	RILPL2	SYNJ2BP	VAPB
APOB	CLIP2	EP300	IFT122	MTDH	PHPT1	RIN1	SYNJ2BP-COX16	VMP1
APP	CLN6	EPB41L1	IGF2BP1	MTERF3	PIK3C2B	RNPEP	TACC2	VPS11

APRT
AQR
ARF1
ARF3
ARF5
ARF6
ARFGEF2
ARFIP2
ARGLU1
ARHGAP5
ARHGDIB
ARHGEF19
ARHGEF38
ARHGEF7
ARID3C
ARIH1
ARIH2
ARL1
ARL2
ARL3
ARL6IP1
ARL6IP5
ARMC7
ARPIN
ARRB1
AS3MT
ASH1L
ASL
ASNA1
ATAD1
ATE1
ATG16L1
ATL2
ATL3
ATP1A3
ATP5F1
ATP5I
ATP5S
ATP6V0A1
ATP6V1C1
ATP6V1F
ATP9B
ATPAF1
ATR
ATXN2L
ATXN7
ATXN7L2
BAG2
BAG3
BAIAP2
BAX
BCAP31
BCCIP
BCL2L10
BECN1
BHLHB9
BICD1
BIN2
BLOC1S4
BLVRB
BNIP1
BPIFB6
BRD2
BRD3
BRINP1
BRIP1
BRIX1
CLNS1A
CLPTM1
CLPX
CLTA
CLTB
CMYA5
CNN3
CNOT1
CNOT4
CNPY4
CNTLN
CNTN5
CNTNAP3B
COG4
COG7
COL18A1
COL1A1
COL4A2
COL4A3BP
COL7A1
COLEC12
COPB1
COPE
COPS7A
COPS8
COX5B
COX6B1
CPNE2
CPNE3
CPNE4
CPNE5
CPNE6
CPNE7
CPNE8
CPNE9
CPOX
CPSF1
CPSF2
CPSF3
CPSF3L
CPT1A
CPT1C
CPT2
CRELD2
CRTAP
CRY1
CRY2
CRYBB3
CSNK2A2
CSRP1
CTNNBL1
CTNND2
CTSB
CTSD
CTSL
CTSS
CTSV
CTTN
CTTNBP2
CWF19L1
CYB5A
CYP2J2
CYP4X1
DAAM2
DAB1
DAB2IP
DAD1
EPHA2
EPM2AIP1
ERC1
ERC2
ERH
ERMP1
ERVPABLB-1
ESPN
ESPNL
EWSR1
EXD3
EXOSC9
EYS
FAF2
FAM104B
FAM114A1
FAM114A2
FAM129A
FAM13A
FAM161B
FAM162A
FAM170B
FAM188A
FAM50A
FAM50B
FAM98A
FAM98B
FANCE
FBXL2
FBXL20
FBXL8
FBXO39
FCAMR
FCF1
FCRLB
FECH
FEN1
FERMT2
FILIP1L
FKBP2
FKBP3
FLVCR1-AS1
FN3KRP
FNBP1
FNIP1
FNTB
FRMD4B
FRMPD2
FRYL
FSTL4
FTO
FTSJ3
FUBP3
FUNDC2
FUS
FUSIP1
FXN
GAA
GABBR2
GALNT10
GALNT11
GALNT7
GAS2
GAS8
GCLC
GCLM
GCNT1
IGHMBP2
IGHV3-15
IGHV3-72
IGHV3-73
IL26
IL6ST
ILVBL
IMMP2L
IMMT
IMPA2
INA
INCENP
INTS3
INTS6
INTS7
INTS9
IPO4
IPO8
IQCA1
IQCD
IQGAP3
IREB2
IRS4
ISCU
ISOC1
ISY1
ITCH
ITGA5
ITGAD
ITGAL
ITGB1
ITIH3
IVD
JAK3
JAKMIP1
KALRN
KANK4
KAT6B
KCMF1
KCNAB3
KCNK12
KCTD9
KIAA0196
KIAA1033
KIAA1549
KIAA1551
KIAA1671
KIF11
KIF13A
KIF15
KIF1B
KIF1Bbeta
KIF21A
KIF2A
KIF3A
KIF3B
KIF6
KLC1
KLHL3
KMO
KPNA2
KRR1
KRT10
KRT2
LAMB2
LAMTOR2
LANCL1
MTMR2
MTMR4
MTOR
MTTP
MTX1
MTX2
MUC16
MUSK
MYCBP2
MYDGF
MYH10
MYH15
MYH3
MYL1
MYL12A
MYL12B
MYNN
MYO15A
MYO18A
MYO5A
MYO6
NAA25
NAA40
NAA50
NAE1
NAGK
NAGLU
NANS
NAP1L1
NAP1L4
NAPA
NAPG
NAT10
NAT16
NAV2
NAV3
NCAPD2
NCDN
NCK1
NCOR2
NCSTN
NDRG3
NDST1
NDST2
NDST3
NDST4
NDUFA5
NDUFAB1
NDUFB2
NDUFB6
NDUFB7
NDUFB9
NDUFS2
NDUFS3
NDUFS7
NDUFS8
NDUFV1
NEDD8
NEDD8-MDP1
NEMF
NEXN
NFIB
NFU1
NFYC
NIT2
NMD3
NME1
PIK3CA
PIN1
PIWIL3
PKD1
PKD1L1
PKN1
PLA2G4A
PLAA
PLBD2
PLCB3
PLCD1
PLCG1
PLEKHA6
PLEKHH1
PLOD2
PLPPR3
PLXNB1
PMM1
PMM2
PMS2P2
PNP
PNPLA7
POC5
POLA1
POLA2
POLDIP2
POLR1E
POLR2G
POLR2H
POM121C
PON3
POTEJ
PPFIA1
PPFIBP2
PPIC
PPIG
PPIL2
PPM1D
PPP1R10
PPP1R12A
PPP1R12B
PPP1R35
PPP2R1B
PPP2R2A
PPP2R2B
PPP2R2D
PPP4R1
PPP4R1L
PPWD1
PREX2
PRKAA1
PRKAG1
PRKAR2A
PRKCA
PRKCB
PRKCE
PRKCH
PRKCQ
PROSC
PRPF3
PRPF31
PRPF4
PRPF6
PRPH
PRPS1L1
PRPS2
PRPSAP1
ROBO2
RPA1
RPA2
RPA3
RPL27A
RPL36A-HNRNPH2
RPL37AP8
RPL39
RPL39P5
RPP25L
RPP30
RPRD1B
RPS6KA5
RPTOR
RPUSD2
RRAGA
RRAGB
RRAGC
RRAGD
RRM2
RRP1
RRP12
RRP9
RSL24D1
RSPH3
RTCA
RTKN2
RTN4
RXFP4
RXRA
RXRB
RXRG
S100A10
SALL1
SCCPDH
SCFD1
SCN8A
SCP2
SCYL2
SDHD
SEC22B
SEC24B
SEC24D
SEC61A2
SEC61B
SEC63
SELENBP1
SELT
SEMA3B
SEMA6B
SEP15
SEPT12
SEPT14
SEPT8
SERBP1
SERGEF
SERINC5
SERPINA3
SETD3
SETD7
SF3B2
SF3B4
SF3B6
SFSWAP
SGK1
SH2D4A
SH3GL1
TANC2
TANK
TARBP1
TAS2R39
TATDN1
TAX1BP3
TBC1D2
TBC1D4
TBKBP1
TBL1X
TBL1Y
TBL3
TBRG4
TCEA2
TCHH
TDP1
TEC
TEP1
TFEB
TFG
TFR2
TFRC
TGM1
TGM2
TGM5
THADA
THOC3
TIMM23
TIMM23B
TJP1
TM9SF1
TMED7
TMEM11
TMEM167A
TMEM192
TMEM2
TMEM5
TMEM79
TMLHE
TNC
TNPO2
TNRC6A
TNRC6B
TNS2
TOE1
TOMM22
TOMM34
TOMM40
TOP3A
TOR1B
TPD52
TPD52L2
TPGS1
TPM1
TPP1
TRA2A
TRA2B
TRAP1
TRAPPC11
TRIB3
TRIM24
TRIM3
TRIM32
TRIM33
TRIM39
TRIM39-RPP21
TRIM47
VPS18
VPS25
VPS26A
VPS26B
VPS29
VPS37B
VPS53
VRK1
VWA5B2
VWA8
WAPL
WDR36
WDR37
WDR43
WDR48
WDR5
WDR6
WDR60
WDR61
WDR75
WDR77
WDR81
WDR82
WDR87
WIPI1
WWP1
WWP2
XAB2
XRN2
XRRA1
YKT6
YRDC
YTHDC2
YTHDF1
YTHDF2
YTHDF3
YY1
YY2
ZADH2
ZBED4
ZBTB10
ZCCHC2
ZFC3H1
ZFP42
ZFP90
ZFP91
ZFP91-CNTF
ZFR
ZMPSTE24
ZNF233
ZNF462
ZNF662
ZNF83
ZNF853
ZPR1
ZW10

BRK1	DCAF13	GCSH	LARP7	NOC2L	PRR23A	SH3GL2	TRIO
BRWD1	DCTN1	GET4	LCLAT1	NOP10	PRR23B	SH3GLB1	TRIOBP
BYSL	DCTN2	GHITM	LCMT1	NOXA1	PRR36	SH3GLB2	TRIP11
C11orf95	DCTN3	GLG1	LDHAL6A	NPC2	PRRC2A	SHISA6	TRIP12
C14orf159	DCTN4	GLRX5	LGALS3BP	NPDC1	PRSS1	SIN3A	TRMT10A
C15orf38-AP3S2	DCUN1D1	GLUL	LGALSL	NPEPL1	PRSS23	SKIV2L2	TRMT13
C15orf56	DDX18	GLYCTK	LIG1	NPTN	PSAP	SLC12A9	TRMT61A
C16orf62	DDX42	GMPPA	LILRA6	NRDC	PSEN1	SLC15A3	TRNT1
C18orf65	DDX47	GMPPB	LIMS1	NRP2	PSEN2	SLC25A11	TRPM2
C19orf70	DDX49	GNA11	LIMS4	NSA2	PSIP1	SLC25A20	TRPM8
C1orf106	DDX54	GNA13	LIN7B	NSMCE4A	PSMB7	SLC25A27	TSC22D1
C1orf167	DDX6	GNA14	LMTK3	NT5C	PSMD7	SLC38A11	TSNAX
C21orf2	DEK	GNAI1	LNPEP	NTMT1	PSMG1	SLC39A12	TTC25
C22orf31	DENND3	GNAI2	LOC100996720	NUCB1	PSMG3	SLC44A1	TTC31
CAB39	DENND4B	GNAQ	LOC102724159	NUCB2	PSPC1	SLC6A11	TTC34
CACNA1H	DERL1	GNAS	LONP2	NUCKS1	PTGR2	SLC6A8	TTLL12
CACNA1S	DGCR6	GNE	LONRF3	NUDCD1	PTGS1	SLK	TTLL13P
CACNB3	DGCR6L	GNL1	LPCAT1	NUDT21	PTMA	SLU7	TUBA4A

Table S6 Transcription factors regulating *MZF1* revealed by UCSC

ATF1	FOSL2	NFYB	STAT3
ATF2	FOXA1	NR3C1	TAF1
ATF3	FOXP2	NRF1	TBP
BACH1	GABPA	PAX5	TCF12
BHLHE40	IRF1	PBX3	TCF3
CEBPB	IRF3	RELA	TCF4
CTCF	JUN	REST	TCF7L2
CTCFL	JUND	RFX5	USF1
E2F1	KDM5B	RUNX3	USF2
E2F4	MAX	SIX5	YY1
EGR1	MAZ	SP1	ZNF143
ELF1	MXI1	SP2	ZNF263
ELK1	MYC	SP5	
ETS1	NFIC	SPI1	
FOS	NFYA	SRF	

Table S7 MZF1-uPEP expression in human NB tissues

Group	Total number	MZF1-uPEP expression				Positive rates (%)	*P*-Value
		−	+	++	+++		
Age							
<1 year	20	5	6	5	4	75.0	0.038
⩾1 year	22	15	4	2	1	31.8	
Differentiation							
Well differentiated	8	0	2	3	3	100.0	
Poorly differentiated	28	15	7	4	2	46.4	0.024
Undifferentiated	6	5	1	0	0	16.7	
MKI							
<200	17	5	3	5	4	70.6	0.037
>200	25	15	7	2	1	40.0	
INSS stages							
Stage 1-2	14	2	3	5	4	85.7	
Stage 3-4	20	15	4	1	0	25.0	0.007
Stage 4S	8	3	3	1	1	62.5	
MYCN amplification							
No	34	15	8	6	5	55.8	0.627
Yes	8	5	2	1	0	37.5	

MZF1-uPEP, MZF1 uORF-encoded peptide; MKI, mitosis karyorrhexis index; INSS, international neuroblastoma staging system.

Table S8 YY1 and MZF1 expression in human NB tissues

Group	Total number	YY1 expression				Positive rates (%)	*P*-Value	MZF1 expression				Positive rates (%)	*P*-Value
		−	+	++	+++			−	+	++	+++		
Age													
<1 year	20	11	4	4	1	45.0	0.011	14	3	2	1	30.0	0.001
⩾1 year	22	2	9	6	5	90.9		2	8	8	4	90.9	
Differentiation													
Well differentiated	8	6	1	1	0	25.0		4	2	2	0	50.0	
Poorly differentiated	28	7	11	9	1	75.0	<0.001	12	9	7	0	57.1	<0.001
Undifferentiated	6	0	1	0	5	100.0		0	0	1	5	100.0	
MKI													
<200	17	9	5	1	2	47.1	0.037	11	3	2	1	35.3	0.033
>200	25	4	8	9	4	84.0		5	8	8	4	80.0	
INSS stages													
Stage 1-2	14	9	3	2	0	35.7		10	3	1	0	28.6	
Stage 3-4	20	2	7	6	5	90.0	0.042	3	5	8	4	85.0	0.025
Stage 4S	8	2	3	2	1	75.0		3	3	1	1	62.5	
MYCN amplification													
No	34	10	12	9	3	70.6	0.140	14	9	8	3	58.8	0.598
Yes	8	3	1	1	3	62.5		2	2	2	2	75.0	

YY1, Yin Yang 1; MZF1, myeloid zinc finger 1; MKI, mitosis karyorrhexis index; INSS, international neuroblastoma staging system.

Table S9 Correlation between the expression of MZF1-uPEP, YY1, and MZF1

	MZF1 expression			
	Low	High	*R*-value	*P*-value
**MZF1-uPEP expression**				
Low	15	15	-0.471	0.002
High	12	0		
**YY1 expression**				
Low	23	3	0.643	<0.001
High	4	12		

MZF1, myeloid zinc finger 1; MZF1-uPEP, MZF1 uORF-encoded peptide; YY1, Yin Yang 1; Pearson's correlation coefficient was applied to determine the expression correlation.
